# 7,8‐Dihydro‐8‐oxoguanosine Lesions Inhibit the Theophylline Aptamer or Change Its Selectivity

**DOI:** 10.1002/cbic.201900684

**Published:** 2020-01-30

**Authors:** Courtney Kiggins, Austin Skinner, Marino J. E. Resendiz

**Affiliations:** ^1^ Department of Chemistry University of Colorado Denver 1151 Arapahoe Street, Science Building Room 4145 Denver CO 80204 USA; ^2^ Present address: Department of Chemistry U.S. Air Force Academy 2355 Fairchild Drive USAF Academy Colorado Springs CO 80840 USA

**Keywords:** 8-oxoG, aptamers, oxidation, RNA structures, thermophoresis

## Abstract

Aptamers are attractive constructs due to their high affinity/selectivity towards a target. Here 7,8‐dihydro‐8‐oxoguanosine (8‐oxoG) has been used, due in part to its unique H‐bonding capabilities (Watson–Crick or Hoogsteen), to expand the “RNA alphabet”. Its impact on the theophylline RNA aptamer was explored by modifying its binding pocket at positions G11, G25, or G26. Structural probing, with RNases A and T_1_, showed that modification at G11 leads to a drastic structural change, whereas the G25‐/G26‐modified analogues exhibited cleavage patterns similar to that of the canonical construct. The recognition properties towards three xanthine derivatives were then explored through thermophoresis. Modifying the aptamer at position G11 led to binding inhibition. Modification at G25, however, changed the selectivity towards theobromine (*K*_d_≈160 μm), with a poor affinity for theophylline (*K*_d_>1.5 mm) being observed. Overall, 8‐oxoG can have an impact on the structures of aptamers in a position‐dependent manner, leading to altered target selectivity.

## Graphical abstract

**Oxidation of RNA in aptamers—8‐oxoG**: Incorporation of the 8‐oxoG unit within the aptamer for theophylline altered its selectivity and affinity. Modification at position G25 changed the selectivity in favor of theobromine, whereas the G11‐modified aptamer experienced drastic structural changes that resulted in inhibited recognition of three xanthine derivatives.

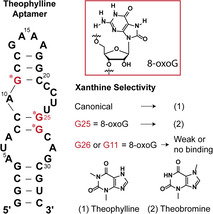

## Introduction

An aptamer is the smallest fragment of a biopolymer that is able to recognize a particular target with high affinity and specificity, often with a sub‐micromolar/sub‐nanomolar dissociation constant.^1^ These constructs display recognition of a wide array of different molecules and their targets range from large biopolymers such as proteins^2^ to small molecules^3^ or ions.^4^ In addition, their small size, high flexibility, and ease of manufacture make them attractive candidates for various applications, including as sensors, materials, or replacements for antibodies.^5^ The potential for functionalization of nucleic acid aptamers with a plethora of groups at various positions has enabled the development of these systems into functional structures with promise in therapeutic applications.^6^ However, a potential disadvantage in comparison with their analogous protein structures (antibodies) is the lack of diversity in the structural set, which is restricted to the four nucleobases (G, A, C, U).^7^ This has prompted efforts to diversify the nature of the nucleobases with other groups.^8^ Of note are examples in which a selection process led to chemically modified aptamers with dissociation constants in the nanomolar to sub‐picomolar range, and with potential for therapeutic applications, with selectivity towards biologically relevant targets such as cancer cell lines, aspartyl protease β‐secretase 1 (BACE1), proprotein convertase subtilisin/kexin type 9 (PCSK9), vascular endothelial cell growth factor‐165 (VEGF‐165), or interferon‐γ (IFN‐γ).^9^ Specifically, the modified nucleobases that were used in these works include 7‐(thiophen‐2‐yl)imidazo[4,5‐*b*]pyridine, 5‐chlorouracil/7‐deazadATP mixtures, or C5‐modified pyrimidine systems containing hydrophobic moieties such as naphthyl, phenyl, or morpholino groups. We are interested in probing the H‐bonding capabilities of modified nucleobases to control the function of aptamers and to diversify the “toolkit” to generate constructs with distinct selectivity and, ideally, increased affinity.

**Scheme 1:**
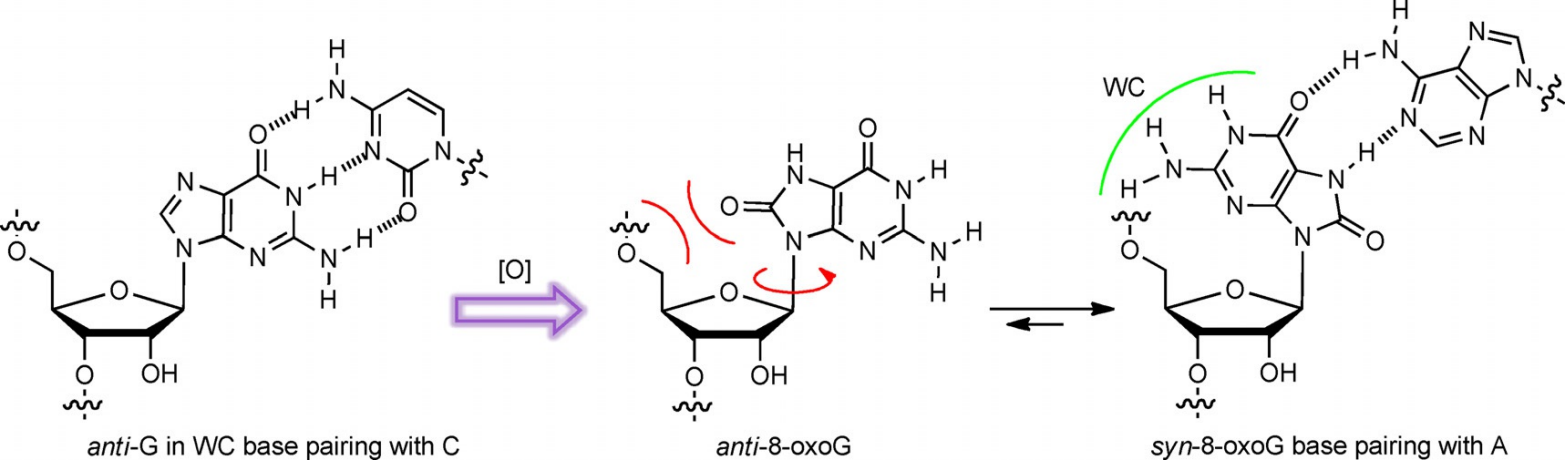
Oxidation of G at the C8‐position leads to 8‐oxoG. The group at this position experiences steric hindrance with the C5′‐hydrogen atoms, and this induces a conformational change to the *syn* isomer.

We opted to explore a modification that has been widely studied in other contexts: 7,8‐dihydro‐8‐oxoguanosine (8‐oxoG). Of the many oxidative lesions that have been characterized in DNA or RNA, purine‐derived modifications are expected to be the most abundant [redox potential trend=G<A<C<U].^10^ Two important features that make 8‐oxoG a unique structural building block are: 1) its preference to undergo an *anti*→*syn* conformational change around the glycosidic bond, and 2) the distinct H‐bonding patterns arising from each of these isomers (or, put another way, its ability to form stable base pairs with cytosine or adenine, Scheme 1). The nature of the conformational change to the *syn* isomer is known to be independent of solvent (DMSO or water) or pH (5–8) and is due to steric hindrance between the C5′‐position and the atom at the C8‐position.^11^ Thus, it is expected that, in a single‐stranded RNA, this will be the preferential conformation. However, because both faces can be involved in H‐bonding, this context may vary for cases in which 8‐oxoG is involved in intra‐ or intermolecular interactions, such as in its folding to other secondary structures,^12^ or in H‐bonding with other biopolymers.^13^ These factors are known to present a challenge from a biological point of view, if the modification is generated in vivo, and have been characterized in monomers^14^ and in oligonucleotides (ONs).^15^ On the other hand, this behavior can be attractive and promising from a design perspective: that is, in the use of 8‐oxoG as an ON building block to target small molecules or proteins of interest. The 8‐oxoG unit offers the possibility of altered function, due to its inherent capability to generate H‐bonding networks that are distinct from those generated by its canonical analogues, yet structurally similar. This property has been explored in the design of compounds that fit the H‐bonding pattern of 8‐oxoG for its detection,^16^ to control DNA structure,^17^ in the formation of supramolecular helices with potential uses for electronic biodevices,^18^ or in the use of C8‐substituted guanosine analogues to understand biological mechanisms.^19^ Of note is an example in which a DNA aptamer was designed to detect 8‐oxoG through interactions similar to those shown in Scheme 1.^20^ In addition, the presence of 8‐oxoG has also been shown to affect the overall structure of DNA and to lead to its deformation.^21^ These examples provide evidence that, although the effects of this oxidative modification on RNA have not been explored in detail, there is great potential in using it as a handle to control the structure and function of RNA.

To probe for the impact that 8‐oxoG has on the structure and function of RNA, the aptamer for theophylline was used as model. This construct was originally selected with the development of SELEX over two decades ago.^22^ Since then, it has been used as a model system for various applications,^23^ such as to explore new sensing methodologies,^24^ to establish theoretical models,^25^ or to detect theophylline in serum.^26^

**Scheme 2:**
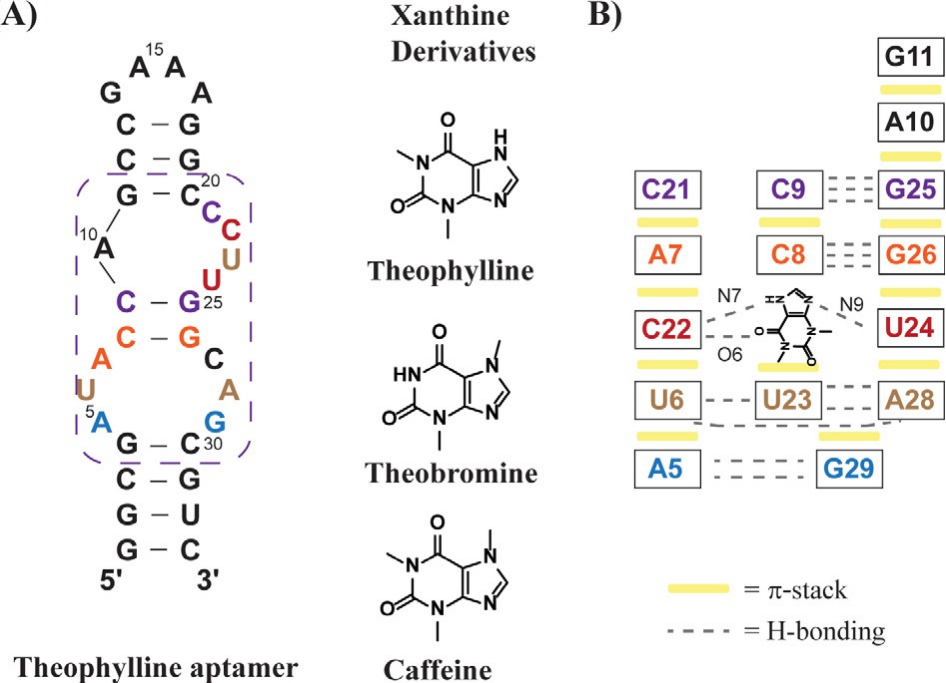
A) Sequence of theophylline aptamer and structures of xanthine derivatives. B) Intrastrand/intermolecular interactions (color‐coded) that are directly involved in theophylline recognition.

The construct is an RNA strand, 33 nt in length, with high selectivity for theophylline^27^ (Scheme 2). This represents a good model because of existing detailed knowledge on the interactions between the RNA and the small molecule. Specifically, it is known that four guanosine units (G4, G25, G26, G29) are involved in the recognition of theophylline whereas three more are present in the vicinity of the active site (G11, G19, and G31), thus making these positions attractive candidates for probing the impact that the 8‐oxoG lesion has on structure and small‐molecule recognition. This aptamer is able to recognize the small molecule over other targets, containing minor structural differences, with up to 10 000‐fold increased affinity.^28^ It has been established, for example, that the canonical aptamer can recognize theophylline (1,3‐dimethylxanthine) over theobromine (3,7‐dimethylxanthine), which differs in the position of a single methyl group, or caffeine (1,3,7‐trimethylxanthine), which contains an additional methyl group (Scheme 2 A).

In brief, three levels of scaffolding provide support for the aptamer's binding pocket (Scheme 2 B), which is composed of 14 nucleotides that form a platform/lower loop, as well as a ceiling, that fits the theophylline and recognizes it through hydrogen‐bonding interactions with C22 and U24. The aptamer contains an S‐turn and maintains a shape that brings the lower and upper loops of the conserved region into proximity to intercalate, thus generating the binding pocket.^29^ Specifically, U23 H‐bonds to A28, due to the S‐turn in its tertiary structure, and this allows a base triple interaction with U6 to form the floor of the binding pocket, whereas a base triple interaction between A7, C8, and G26 provides the “ceiling” of the binding pocket. One side of the recognition site is generated through π–π stacking interactions between C21, A7, C22, and U6, whereas the other side is formed by G26 intercalation between G25 and U24, thought to stabilize the sharp bend in the tertiary structure to allow C22 to intercalate between U6 and A7. In addition, providing further stabilization of the binding core, A10 π‐stacks with G25 and G11. An important factor to note is that A7 is displaced from its typical A‐form helical position, thus generating space that facilitates binding to the target. With regard to the high affinity towards theophylline, C22 and U24 are involved in its discrimination from other xanthine derivatives through H‐bonding interactions with N7−H and the O6 carbonyl group of the small molecule.

## Results

**Figure 1:**
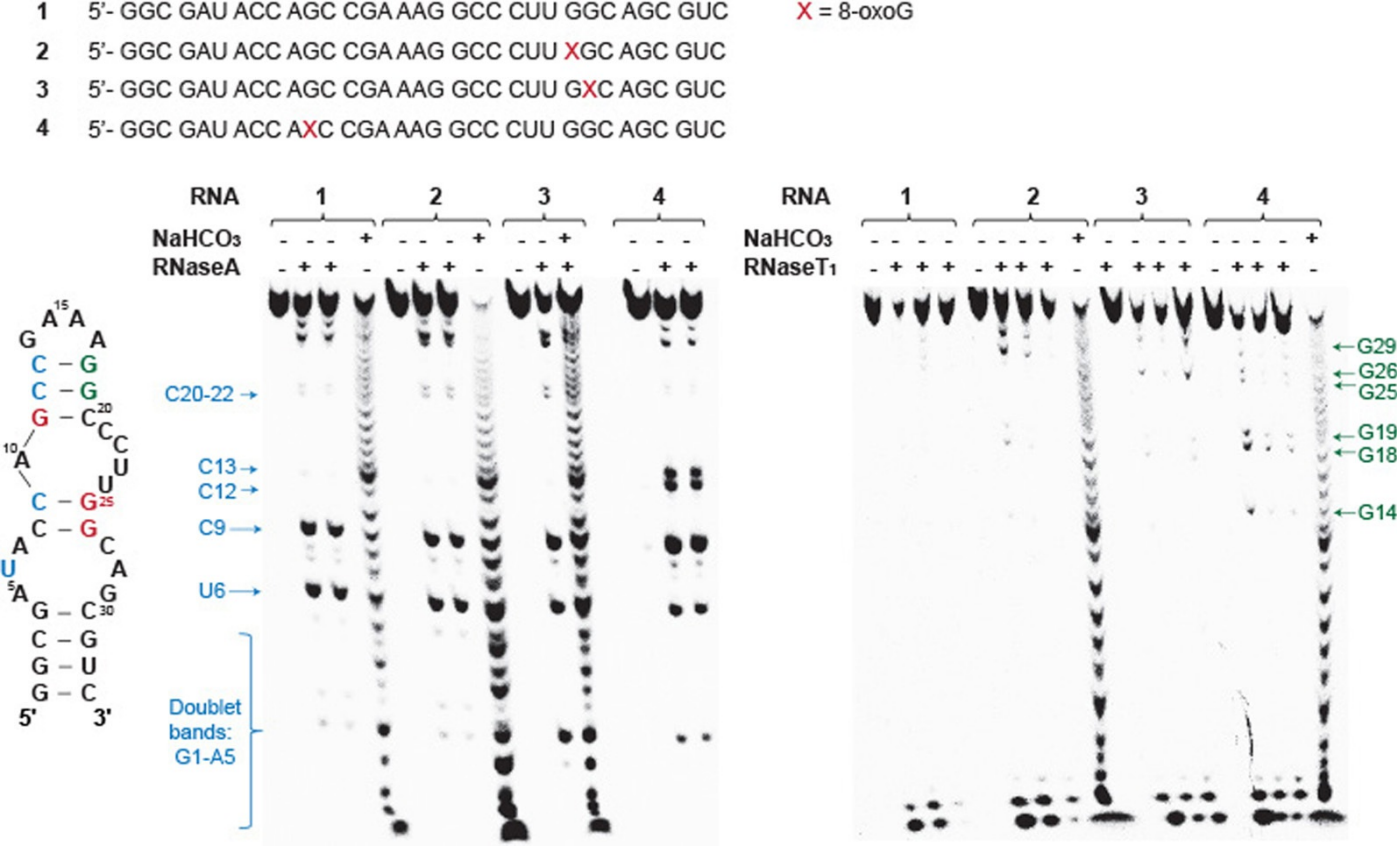
Sequences of RNA strands **1**–**4** (top) and folding of canonical strand **1**, together with 20 % denaturing PAGE of RNAs **1**–**4** after treatment with (left) RNase A or (right) RNase T1. A hydrolysis ladder (NaHCO_3_) was used for fragment assignment. Pyrimidine and guanine rings in which cleavage was observed are highlighted in blue and green, respectively.

We set out to explore the impact of the 8‐oxoG unit on the theophylline RNA aptamer and incorporated the lesion into ONs of RNA via solid‐phase synthesis. The RNA construct (strand **1**), 33 nucleotides in length, was modified with 8‐oxoG at positions G25, G26, and G11, chosen on the basis of their roles in target recognition within the binding pocket, to yield RNA strands **2**, **3**, and **4** respectively (Figure 1). Importantly, the construct for which more structural information is available was chosen.^28^

To understand the impact of the modification on the structure, all strands were radiolabeled and treated with RNase A, which cleaves single‐stranded RNAs at both pyrimidine and 8‐oxoG sites into fragments containing 5′‐OH and 3′‐phosphate ends.^30^ The results were then compared with those obtained with canonical strand **1**. As shown in Figure 1 (left), RNA strands **1**–**3** displayed hyperreactivity at positions U6 and C9, and to a lower extent some cleavage in the C20–C22 region (in the cases of modified strands **2** and **3**), thus suggesting that these positions are in environments available for ribonuclease access and not restricted to H‐bonding interactions with other nucleobases. Cleavage at position C9 was unexpected; however, this marks the beginning of the internal loop (4×1), thus presumably making it more accessible. Interestingly, incorporation of 8‐oxoG at positions G25 or G26 gave rise to similar cleavage patterns, thus suggesting that modification at these sites does not affect the overall structure of the aptamer, or that these positions are cleaved because they are also exposed in another structural arrangement. The only observed difference was that cleavage around the C20–C22 region was increased slightly in the case of RNA **3** (G26‐modified), thus indicating that interactions amongst nucleobases in this section of the construct might be altered. The most striking difference was observed in the case of strand **4**, modified at position G11, which displayed prominent cleavage at additional positions (C12 and C13), consistent with disruption of the stem in the upper hairpin and exposure of these nucleobases to the ribonuclease. Assignment of the bands was carried out by comparing the results against a hydrolysis ladder (NaHCO_3_, pH 9.1). The ladder works through cleavage of every nucleobase from the 3′‐end, thus allowing a band to appear at every position of the aptamer. A pattern in which doublet bands are observed in cases of shorter fragments is consistent with the formation of the 3′‐phosphate, along with the corresponding cyclic phosphate derivative that has been characterized/observed previously.^31^

To complement these observations, experiments were also carried out with RNase T1, a ribonuclease that specifically cleaves at all G‐sites in a single‐stranded context. Interestingly, the only aptamer that showed some hypersensitivity to this ribonuclease was aptamer **4**, which displayed cleavage at positions G14, G18, and G19. Consistent with the results obtained in the case of the RNase A cleavage, this observation points to disruption of the upper hairpin within the construct. Although the intensity of the cleavage bands was weak, the pattern was reproducible. Furthermore, in agreement with alteration at the binding site, cleavage at position G29 took place in the case of RNA strand **2**, whereas position G25 (G26 was ruled out because of the reported inability of RNase T1 to cleave at 8‐oxoG sites) was accessible for ribonuclease activity in the case of modified aptamer **3**.

In an attempt to interpret these changes and to explain the reactivity patterns, the UNAFold server was used to explore possible changes in structure (Figures S5–S8 in the Supporting Information).^32^ As expected, the model for the canonical RNA **1** matched relatively well with the known structure, with the only exception being the prediction of an A11:U24 base pair (Figure S5). Because 8‐oxoG is not available in this hybridization package, we reasoned that substituting the modification with uridine could provide a valid model, on the basis of the H‐bonding similarities between *syn*‐8‐oxoG and U. However, this approach predicted structural changes in the case of RNA strands **2**/**3** (modified at G25 and G26) that do not explain the observed RNase A cleavage patterns (Figures S6 and S7). The same analysis was carried out by substituting G11 with U, which resulted in disruption of the hairpin stem in this region but did not fully explain the RNase A cleavage pattern at positions C12 and C13. Overall, substitution of U in place of 8‐oxoG did not provide a good model in this context.

**Scheme 3:**
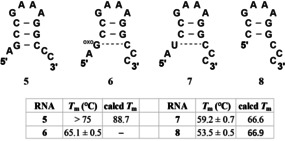
Sequences of hairpins **5**–**8** mimicking the upper scaffold of the aptamer and their corresponding *T*_m_ values (experimentally measured and calculated with UNAFOLD). Conditions: RNA 3.5 μm, NaCl 1 mm, MgCl_2_ 5 mm, Na_2_P_2_O_7_ 10 mm, pH 7.2.

To assess the impact that 8‐oxoG might have in the upper stem, RNA strands **5** and **6**, containing G or 8‐oxoG (Scheme 3), were prepared in order to mimic this structural motif. The substitution was incorporated at position 2 of this construct and all strands displayed bands consistent with formation of a hairpin (bands with positive and negative ellipticity at 260 and 210 nm, respectively). Thermal denaturation transitions were obtained by measuring the hypochromic shift in the dichroic signal at 260 nm as a function of temperature and were independent of concentration, thus suggesting unimolecular transitions. Incorporation of 8‐oxoG, as in strand **6**, resulted in a large thermal destabilization relative to canonical RNA hairpin **5**. This is consistent with other cases in which an oxidative lesion has a large impact on hairpin stability.^33^

To confirm that the 8‐oxoG base pairs with C in this context, hairpins **7** (containing U) and **8** (lacking a nucleotide) were also prepared, and displayed larger thermal destabilization in both cases. This result indicates that 8‐oxoG does interact with C at this position, albeit more weakly than in the case of the corresponding WC pair. The observed trends were in agreement with those predicted by use of UNAFOLD, albeit with values that were consistently lower than those observed experimentally (Figures S9–S11 and Scheme 3, inset). Overall, the difference in the thermal stability between hairpins **5** and **6** (Δ*T*_m_ ≈−9 °C) suggests that positioning 8‐oxoG in the aptamer at this position (G11, Figure 1) leads to a drastic structural change, yet to be fully characterized.

### Small‐molecule binding

**Figure 2:**
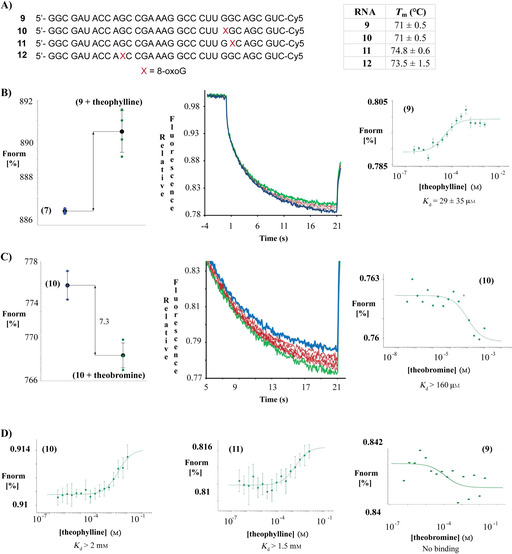
A) Sequences of Cy‐5‐labeled RNAs **9**–**12** and their corresponding thermal denaturation transitions (*T*_m_), obtained in a 10 mm sodium phosphate buffer (pH 7.5) and a 50 mm Tris**⋅**HCl saline (TBS) buffer (pH 7.6). MST binding check, binding traces, and *K*_d_‐fit curve of B) construct **9** with theophylline, and C) construct **10** with theobromine (additional curves are included in Figure S14, displaying the same binding check and *K*_d_ calculation with a different buffer). The blue trace corresponds to unbound RNA, the green trace to RNA bound to the small molecule, and the red traces correspond to the RNA titrated with the small molecule (some curves at different concentrations were omitted for clarity). D) *K*_d_‐fit curve of constructs **10**/**11** with theophylline and **9** (no binding) with theobromine. All curves were obtained in triplicate.

We then explored the impact that the 8‐oxoG lesion has on small‐molecule recognition and used microscale thermophoresis (MST) to establish the selectivity and affinity of each construct towards the three xanthine derivatives. This technique was chosen because it allows for the determination of the dissociation constant (*K*_d_) between an aptamer and its cognate ligand in free solution and with minimal sample consumption.^34^ This technique relies on recording the thermophoretic effect of the aptamer in the absence and in the presence of the target molecule,^35^ and is carried out by measuring changes in fluorescence as the bound/unbound ON migrates across a heat gradient inside a capillary.^36^ To this end, ONs (modified at the same positions as in the previous strands: G11, G25, G26) containing a cyanine‐5 dye (*λ*_abs_=646 nm, *λ*_em_=662 nm) at the 3′‐end were prepared via solid‐phase synthesis to yield RNA strands **9**–**12** (Figure 2 A). To interpret the data from the binding thermophoretic assays, the recommendations from a recent report were followed.^37^ In addition, because interactions between nucleic acids and their targets can be affected by salt and buffer concentrations^38^ we used buffer systems that have been reported previously.28b

Binding checks were performed in the presence of each target to validate interactions between the small molecule and the aptamer, or to rule out aptamers that do not display specific binding; these experiments were carried out by measuring the ON mobility either in the absence or in the presence of high concentrations of the small molecules. As depicted in Figure 2 B (left) the canonical aptamer **9** displayed binding to the expected target, theophylline, whereas no binding occurred in the presence of theobromine or caffeine. Solutions containing the small molecule were then prepared by making sixteen twofold dilutions, to obtain a dissociation constant (*K*_d_) of (29±35) μm, a value consistent with the literature.28b, 29 In addition, isothermal titration calorimetry (ITC) was used to validate binding of the canonical RNA aptamer **1** to theophylline, to obtain a *K*_d_ value of 1.35 μm (Figure S24). Interestingly, modified aptamers **10** and **11** also displayed binding affinity towards theophylline, albeit with affinities approximately two orders of magnitude weaker (*K*_d_>1.5 mm, Figure 2 D). An exact quantity could not be established, due to the low solubility of the small molecules in the buffer system at higher concentrations. The fact that these two aptamers recognize theophylline, to some extent, is not surprising in view of their similar structural characteristics (as observed through enzymatic cleavage experiments). On the other hand, as shown in Figure 2 C, aptamer **10** (modified at G25) displayed a higher binding affinity towards theobromine, with *K*_d_>160 μm (a complete sigmoidal curve for this pair could not be obtained, due to the poor solubility of theobromine), and with no binding of caffeine. This result highlights the ability of 8‐oxoG to have an impact on structure as well as on small‐molecule affinity and selectivity. Furthermore, consistent with large structural changes, aptamer **12** (modified at G11) did not display any binding affinity towards the three xanthine derivatives tested in this work. To provide better understanding of the impact of 8‐oxoG as a function of position, the thermal denaturation transitions were obtained (by CD, Figure S15) to show values for aptamers **9** and **10** that are equivalent, whereas aptamers **11** and **12** have increased thermal stability. As established previously, stabilization of structure can, in some cases, be directly related to decreased affinity in target binding.^12^ The large stabilization observed in the case of aptamer **11** might be due to increased interactions between 8‐oxoG and other nucleobases, arising from extended H‐bonding networks formed from both Watson–Crick and Hoogsteen faces.

Attempts to record changes in structure by CD in the absence and in the presence of the xanthine derivatives did not show any appreciable differences (Figure S23). All aptamers displayed dichroic bands consistent with folding into A‐form duplexes. Although the structural probing experiments indicate that a different structure might be formed in the cases of RNAs **4**/**12** (modified at position G11), the CD spectra showed that the extent of the duplex region is comparable to those of the other modified RNA strands, as well as that of the canonical aptamer (Figure S15).

## Discussion

The impact of 7,8‐dihydro‐8‐oxoguanosine (8‐oxoG) on RNA structure and function was explored by using the aptamer for theophylline as a model. The 8‐oxoG motif was independently incorporated onto ONs of RNA and its structural impact was assessed by electrophoretic analyses and circular dichroism, whereas its function was established by assessing the ability of the modified aptamers to bind three different xanthine derivatives: theophylline, theobromine, and caffeine. Thermophoretic analyses showed that the selectivity was altered in the case of modified aptamer **8** (8‐oxoG at position 25), which displayed *K*_d_ values in the micromolar range for theobromine and in the millimolar range for theophylline.

**Scheme 4:**
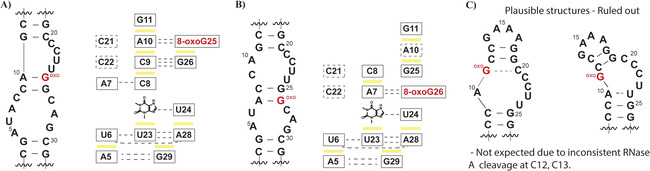
Representations of RNA aptamer modified at position A) G25 (**2**/**8**), or B) G26 (**3**/**9**) and proposed models of how 8‐oxoG might induce a unique tertiary structure that disrupts the binding pocket. C) Plausible structures after modification at G11, showing how 8‐oxoG is likely to have a different, more pronounced, impact on the overall structure.

The results from the RNase A/T1 cleavage studies, along with results obtained from MST experiments, indicate that a modification at G11 has the most drastic impact on the aptamer's structure, whereas modifications at G25 and G26 alter the structure slightly and still allow binding of theophylline. Although RNA **2**/**10** has features that suggest a structure similar to that of the canonical analogue **1**/**9**, based on enzymatic degradation and *T*_m_ analyses, a change in the H‐bonding pattern of 8‐oxoG25 might induce a change in the binding pocket. It is plausible that the *anti*‐to‐*syn* flip for 8‐oxoG25 allows it to hydrogen bond with A10, in turn allowing the formation of WC base pair G26:C9, breaking interactions with C8, which ultimately disrupts the ceiling for the theophylline binding pocket (Scheme 4 A). C8 is necessary for the canonical aptamer's high‐affinity interaction with theophylline. The 8‐oxoG25 and A10 base pairing also disrupts the π‐stacking interaction between G25 and A10; this could then destabilize the binding core and might displace conserved residues such as C21 and C22. The large increase in *K*_d_ is also consistent with a disruption in the binding pocket. The slight degradation seen at G25 could indicate that the H‐bond to A10 is relatively weak. Furthermore, the higher affinity towards theobromine can be explained by the displacement of C22, which is integral in discriminating for theophylline. The conservation of U24 does not provide the ability to discriminate theophylline from xanthine derivatives because N9 is the same in both theophylline and theobromine, and this could thus explain why this aptamer can bind to theobromine.

The results obtained with RNAs **3**/**11**, modified at position 26, showed an increase of over 100‐fold in *K*_d_ towards theophylline, and a lack of recognition for theobromine or caffeine. It is possible that, as depicted in Scheme 4 B, interactions with U6, which is pivotal in forming the floor of the binding pocket, are disrupted by the expected 8‐oxoG26:A7 base pair. This could also displace or prevent U6’s interaction with its corresponding upper‐loop residues and impart stability, evident from a larger *T*_m_ (74.5 °C). As mentioned above, A7 needs the ability to move out of the A‐form helix to create space for the small molecule, and this is unachievable if it is H‐bonding with 8‐oxoG26. This distinct H‐bond might also aid in the formation of a new ceiling for the small molecule, because G25 can now H‐bond with C8. The H‐bond between the 8‐oxoG and A7 might also disrupt the π‐stacking interactions of C21, A7, C22, and U6 by abolishing its ability to intercalate between C21 and C22; this could decrease the interaction between the upper and lower loop residues. Both RNAs **2**/**10** and **3**/**11** retain the ability to bind to theophylline, although weakly, because U24 is still conserved in the core and is potentially not affected by adverse H‐bonding or π‐stacking interactions, while still having an impact on the binding pocket.

We initially hypothesized that modification at G11 (strands **4**/**12**) would not greatly impact small‐molecule recognition. However, this RNA did not bind to any of the xanthine derivatives. This can be explained in terms of the large structural changes observed in the upper loop, evident from the distinct RNase A/T1 cleavage pattern and suggesting that 8‐oxoG11 disrupts the upper scaffolding of the aptamer overall. It is possible that 8‐oxoG11 could be involved in H‐bond interactions with A16 or A17, which would explain why both G18 and G19 are exposed to degradation. In addition, this could result in C21 and C22 being prevented from intercalating with A7 and U6, in turn affecting the role of U24 within the binding core and intercalation between G26 and A28, which would ultimately destabilize the S‐turn that is needed to maintain the integrity of the binding pocket. Attempts to explain the overall structure with the assistance of the UNAFold engine were carried out by substituting 8‐oxoG with U; however, the predicted structures, as well as other possibilities (Scheme 4 C), do not match well with the cleavage patterns. Overall, although the structures of the G11‐modified aptamers were not established, this suggests that the presence of 8‐oxoG at this position has a large impact on the structure and function of this aptamer. Other positions that are potential candidates for G‐to‐8‐oxoG substitution involve G14 and G29. However, two aspects deterred us from probing these positions: 1) they have not been reported to be involved in the recognition of the xanthine derivatives,^29^ and 2) given their proximity to adenosine units, they might potentially generate base‐pair interactions that stabilize the overall structure at the expense of selectivity and/or affinity.^12^

Lastly, another point of relevance relates to the biological impact that oxidative stress has on RNA. Oxidized RNA has been shown to be present in various types of RNA, including rRNA,^39^ mRNA,^40^ or miRNA,^41^ and this also makes riboswitches^42^ prone to oxidative damage. Although this relationship has not been established, it is plausible that such processes might play a role in dysfunction or altered regulatory mechanisms. Thus, understanding the potential structural/functional changes arising from the formation of oxidative lesions is of importance to assess their biological implications.

## Conclusion

The impact that 8‐oxoG has on structure and function, through small‐molecule recognition, was probed with use of the theophylline aptamer as a model. It was shown that the effect of a single 8‐oxoG modification varies as a function of position and that it can result in decreased, or abolished, affinities towards the cognate target molecule, or that it can also change the selectivity of the aptamer towards a different target. Specifically, modification at G25 led to preferential binding of theobromine over theophylline. This result suggests that 8‐oxoG might be usable as a modification in the discovery of aptamers with distinct selectivity/affinity. Although this is a promising strategy, obstacles remain to overcome, including 1) the development of sequencing technologies that would enable a selection process that includes 8‐oxoG in RNA (although recent advances show promise in this respect),^43^ and 2) deeper understanding of the impact of 8‐oxoG on RNA structures within various structural motifs—aspects that are not trivial and that we are working to address.

## Experimental Section

**General**: 8‐OxoG phosphoramidite was synthesized out according to a previous report.^12^ All experiments were carried out in triplicate.

**RNA synthesis**: ONs were synthesized with a 394 ABI DNA/RNA synthesizer and use of CPG supports and 2′‐*O*‐TBDMS phosphoramidites (purchased from Glen Research). 5‐Ethylsulfanyl‐1*H*‐tetrazole (0.25 m) in acetonitrile was used as the coupling reagent, dichloroacetic acid in dichloromethane (3 %) was used for deblocking, a 2,6‐dimethylpyridine/acetic anhydride solution was used for capping, and an iodine/THF/pyridine solution was used in the oxidation step. Coupling times of 10 min were used. ONs were deacetylated/debenzoylated/deformylated and cleaved from the CPG support in the presence of 1:1 aq. methylamine (40 %) and aq. ammonia (40 %) with heating (60 °C, 1.5 h). A mixture of 1‐methylpyrrolidin‐2‐one/triethylamine/HF (3:2:1) was used for removal of the TBDMS groups (60 °C, 1 h), followed by purification by electrophoresis (20 % denaturing PAGE). C18‐Sep‐Pak cartridges were obtained from Waters and used to desalt the purified oligomers with NH_4_OAc (5 mm) as the elution buffer. ONs were dissolved in H_2_O and used as obtained for subsequent experiments. Unmodified ONs were purchased from IDT‐DNA or ChemGenes and, after quantification by UV/Vis, used without further purification. ONs containing a fluorescent Cy‐5 probe at the 3′‐end were synthesized on resin (purchased from Glen Research) containing this moiety and purified as described above.

**RNA characterization**: MS (MALDI‐TOF) was used in the characterization of all modified ONs, with use of C_18_ Zip Tip pipette tips to desalt and spot each ON as follows: 1) wash tip with acetonitrile (50 %, 10 μL×2), 2) equilibrate tip with trifluoroacetic acid (TFA, 0.1 %, 10 μL×2), 3) load tip with sample (typically 100–150 pmol), 4) wash tip with TFA (0.1 %, 10 μL×2), 5) wash tip with water (10 μL×2), 6) elute sample into matrix [10 μL of 2,4,6‐trihydroxyacetophenone monohydrate (25 mm), ammonium citrate (10 mm), ammonium fluoride (300 mm) in aq. acetonitrile (50 %)], and 7) spot directly onto MALDI plate. All analyses were carried out with an ABI 4800 Plus MALDI‐TOF/TOF mass spectrometer in positive mode (see the Acknowledgements).

**UV/Vis spectroscopy**: Concentrations of all ONs were determined by UV/Vis with a PerkinElmer λ‐650 UV/Vis spectrometer and quartz cuvettes (1 cm pathlength). General UV/Vis spectra were also taken with a 1 mm pathlength and 1 μL volumes (Thermo Scientific Nano Drop Nd‐1000 UV/Vis spectrometer). Origin 9.1 was used to plot and normalize spectra of monomers and ONs for comparison.

**Circular dichroism (CD) spectroscopy and thermal denaturation transitions (*T***_**m**_ **)**: CD spectra were recorded at various temperatures (PTC‐348W1 Peltier thermostat) with use of quartz cuvettes with a 1 cm pathlength. Spectra were averaged over three scans (325–200 nm, 0.5 nm intervals, 1 nm bandwidth, 1 s response time) and background‐corrected with the appropriate buffer or solvent. Solutions containing the RNA strands had the following compositions: RNA (1.5 μm), MgCl_2_ (5 mm), NaCl (10 mm), sodium phosphate [pH 7.3 (or other pH values whenever appropriate), 1 mm]. All solutions that had been prepared in order to record thermal denaturation transitions (*T*_m_) were hybridized prior to the recording of spectra by heating to 90 °C followed by slow cooling to room temperature. *T*_m_ values were recorded at 270 nm with a ramp of 1 °C min^−1^ and step size of 0.2 with temperature ranges from 4 to 95 °C. A thin layer of mineral oil was added on top of each solution to keep concentrations constant at higher temperatures. Origin 9.1 was used to determine all *T*_m_ values and to plot CD spectra of RNAs with/without small molecules.

**ON labeling**: T4 polynucleotide kinase (PNK) and γ‐^32^P‐ATP‐5′‐triphosphate were obtained from PerkinElmer. ONs were labeled by mixing PNK, PNK buffer, ATP, DNA, and water (final volume 50 μL) according to the manufacturer's procedure followed by incubation at 37 °C for 45 min. Radiolabeled materials were passed through a G‐25 Sephadex column followed by purification by electrophoresis (20 % denaturing PAGE). The bands of interest (slowest) were extruded and eluted over a saline buffer solution (0.1 m NaCl) for 36 h at 37 °C. The remaining solution was filtered and concentrated to dryness under reduced pressure followed by precipitation over NaOAc and ethanol. Supernatant was removed and the remaining ON was concentrated under reduced pressure and dissolved in water. Activity was assessed with a Beckmann LS 6500 scintillation counter.

**Microscale thermophoresis (MST)**: This technique was used to establish binding affinities and potential for binding with the three xanthine derivatives. RNA strands modified with the Cy‐5 fluorophore at the 3′‐end were used.

**MST—small‐molecule binding studies**: The small‐molecule binding studies were performed with 16 twofold dilutions of each small molecule as can be seen in Table 1. The goal was to measure binding at varying concentrations of small molecule with a constant concentration of RNA to determine the corresponding dissociation constants (*K*_d_). The Monolith NT.115 MST system was used for these studies with concentrations of small molecules as shown in Table 1. Theobromine did not readily dissolve in the buffers used here and was dissolved in DMSO (100 %). This solution was then diluted in the corresponding buffer to achieve solutions with a DMSO content lower than 5 %. Precipitated theobromine was observed in the cases of solutions in which the small molecule was present in the 5 mm range, so experiments were set such that the concentration of theobromine was 1 mm maximum.

**Table 1:** Small molecules used in the binding affinity studies and the corresponding concentration ranges.

Small molecule	Conc. range
theophylline	11.25 mm–343 nm
theobromine	1 mm–30 nm
caffeine	11.25 mm–343 nm

Binding checks were accomplished for all RNAs with each small molecule prior to *K*_d_ determination. The binding checks were carried out to validate binding of the small molecule and aptamer. Eight samples were prepared—four that contained the RNA aptamer and four that contained the RNA and the small molecule. Both the RNA and the small molecule were kept constant at 10 nm and at the highest small‐molecule concentration (e.g., the theophylline concentration in the presence of RNA **9** was 11.25 mm). The prepared mixtures were incubated at room temperature for ≈10 min, captured in a capillary tube (as described below), and placed on the plate, with the RNA sample in rows 1–4 and the RNA/small molecule complex in rows 5–8.

Dissociation constants were obtained as follows. First, 16 microtubes (0.6 mL) were filled with 10 μL of the buffer containing the appropriate small molecule. For theophylline and caffeine, the buffer was 1× TBS [pH 7.5, Tris**⋅**HCl (50 mm), NaCl (150 mm)], whereas for theobromine the buffer was 1× TBS with DMSO (10 %). This was carried out in such a manner that sequential twofold dilution, with respect to the small molecule, was present in each tube. This was followed by addition of RNA (20 nm, previously denatured and hybridized by heating to 90 °C and slow cooling to RT, 10 μL) to each tube to accomplish 10 nm [RNA] (while keeping the content of DMSO at 5 % DMSO max.). The mixtures were then incubated in ice for 20 min in the cases of the mixtures containing theophylline or caffeine and at room temperature in those of the mixtures containing theobromine. Next, ≈10 μL of the mixture was captured in a capillary tube, placed on the plate, and covered with the magnetic strip to prevent movement. Each mixture was withdrawn and handled one at a time, with the solutions containing the highest concentration of small molecule placed at position “1”. The microtube and the capillary were held horizontally to prevent bubble formation, and the capillary was handled by the end of the tube. Each programmed experiment ran for approximately 20 min.

**RNA structural probing**

**RNase A**: A cocktail solution of RNA (3000–5000 counts) in phosphate buffer (pH 5.5, 10 mm) was made. A mixture of the RNA and the enzyme (1:1) was prepared and incubated at RT for 1 h. After incubation, loading buffer [LB: formamide (90 %), EDTA (1 mm), 7 μL] was added, and the mixture of interest (9–10 μL) was added to a denaturing PAGE. For shorter oligomers (<20 nt) a short gel was used. For longer oligomers a long gel was used. The gels were run until the methylene blue dye ran halfway to three quarters of the way down the gel.

**RNase T_1_**: A cocktail solution of RNA (3000–5000 counts) in phosphate buffer (pH 5.5, 10 mm) was made. A mixture of the RNA and the enzyme (1:1) was prepared and incubated at 50 °C for 45 min. After incubation, LB (7 μL) was added, and the mixture of interest (9–10 μL) was loaded onto a denaturing PAGE. For shorter oligomers (<20) a short gel was used. For longer oligomers (>20) a long gel was used. The gels were run until the methylene blue dye ran halfway to three quarters of the way down the gel.

The degradation patterns for all aptamers were compared against a hydrolysis ladder (0.5 m NaHCO_3_, pH 9.1), which was used for band assignment. The ladder works through hydrolysis of every nucleobase from the 3′‐end and was produced by incubating the RNA of interest and the hydrolysis buffer at 90 °C for 12 min (68:32 RNA/NaHCO_3_ ratio, by volume) followed by addition of loading buffer (formamide, 90 %) prior to loading onto gel.

## Conflict of interest

*The authors declare no conflict of interest*.

## Supporting information

As a service to our authors and readers, this journal provides supporting information supplied by the authors. Such materials are peer reviewed and may be re‐organized for online delivery, but are not copy‐edited or typeset. Technical support issues arising from supporting information (other than missing files) should be addressed to the authors.

Supplementary
