# Supplementary material for: 7,8‐Dihydro‐8‐oxoguanosine Lesions Inhibit the Theophylline Aptamer or Change Its Selectivity
Source: Chembiochem. 2020 Jan 30;21(9):1347–55. doi: 10.1002/cbic.201900684 (PMC7297664; doi:10.1002/cbic.201900684)
Supplement: Supplementary file 1 — Supplementary [file CBIC-21-1347-s001.pdf]

CHEMBIOCHEM

Supporting Information

7,8-Dihydro-8-oxoguanosine Lesions Inhibit the Theophylline Aptamer or Change Its Selectivity

Courtney Kiggins,[b] Austin Skinner,[a] and Marino J. E. Resendiz*[a]

cbic_201900684_sm_miscellaneous_information.pdf

Supporting Information Index:

Page **Contents:**

S2-6.....................Figure SI-1 (w/ note), SI-2, SI-3, and SI-4: MALDI-TOF corresponding to RNA strands **1-12**

S7-14...................Figures SI 5 – SI-11: UNAFOLD parameters of ONs **1-8** (8-oxoG substituted with U)

S15…..................Figure SI 12: MST of RNAs **9-12** with theobromine

S16…..................Figure SI 13: MST of RNAs **9-12** with caffeine

S17…..................Figure SI 14. MST of RNA **10** binding with theobromine in a PBS buffer system

S18…..................Figure SI 15. CD spectra and Tm curves corresponding to RNAs **9-12**

S19…..................Figure SI 16. Tm curves corresponding to RNAs **9-12** in TBS buffer, pH 7.6.

S20…..................Figure SI 17-19: CD and T_m curves of RNAs **5** and **6**

S21…..................Figure SI 20-22: CD and T_m curves of RNAs **7** and **8**; and overlay of CDs for strands **5-8**

S22…..................Figure SI 23: CD of RNA **9** with/without theophylline

S23…..................Figure SI 24. ITC of RNA **1** with theophylline and with caffeine

S24…..................References

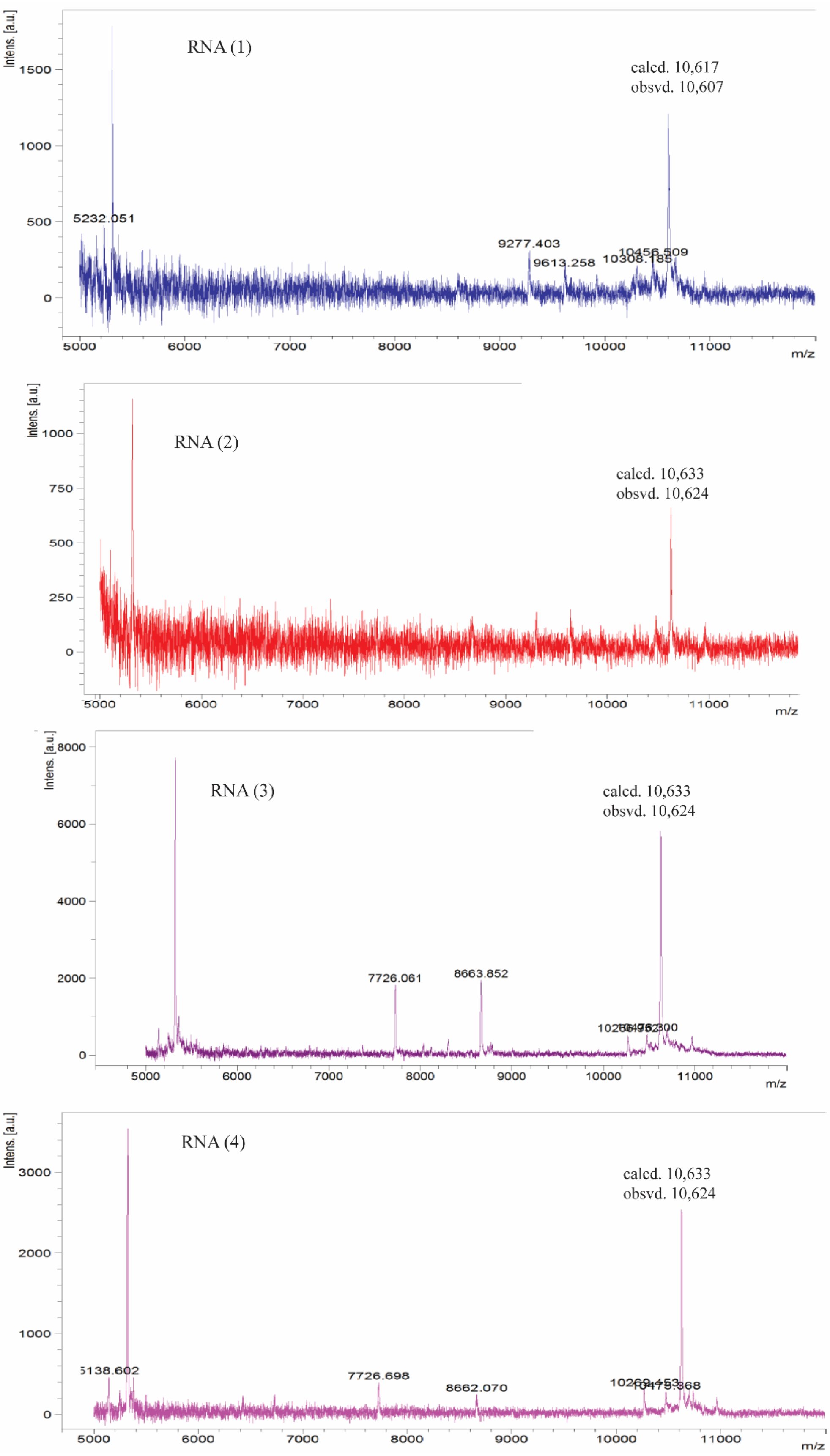

Figure SI 1. MALDI-TOF corresponding to oligonucleotides **1-4**

RNA-(**1**)

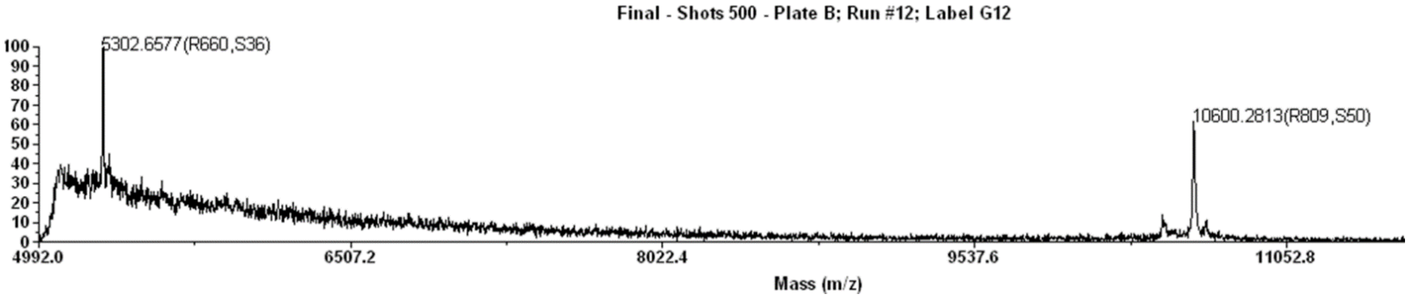

RNA-(**3**)

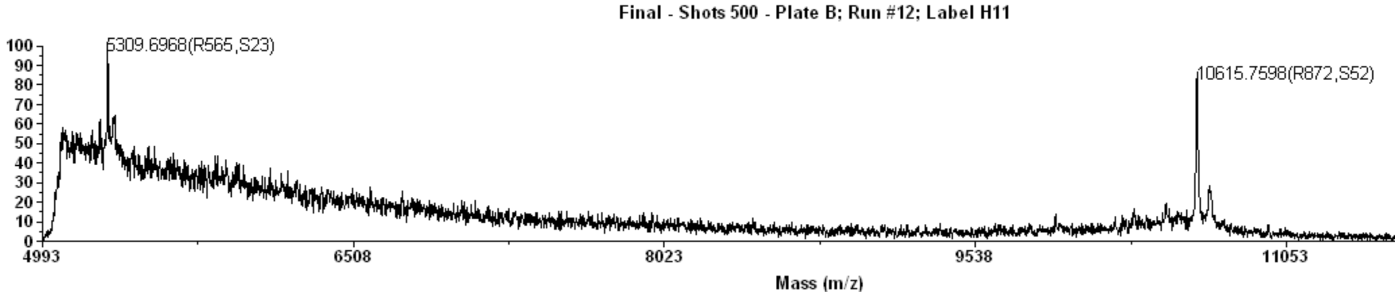

Figure SI 1 (note): As can be observed on the MS-spectra for RNA strands **1**-**4**, there are peaks that correspond to degradation of the corresponding aptamers (particularly RNA **3**). These occurred in the shipping of the sample to the facility at Colorado State University, or upon sample preparation. MALDI-TOF of samples not shipped are shown above and show that the aptamers are pure (however, no calibrant was spotted on this and a difference in the parent peak was recorded).

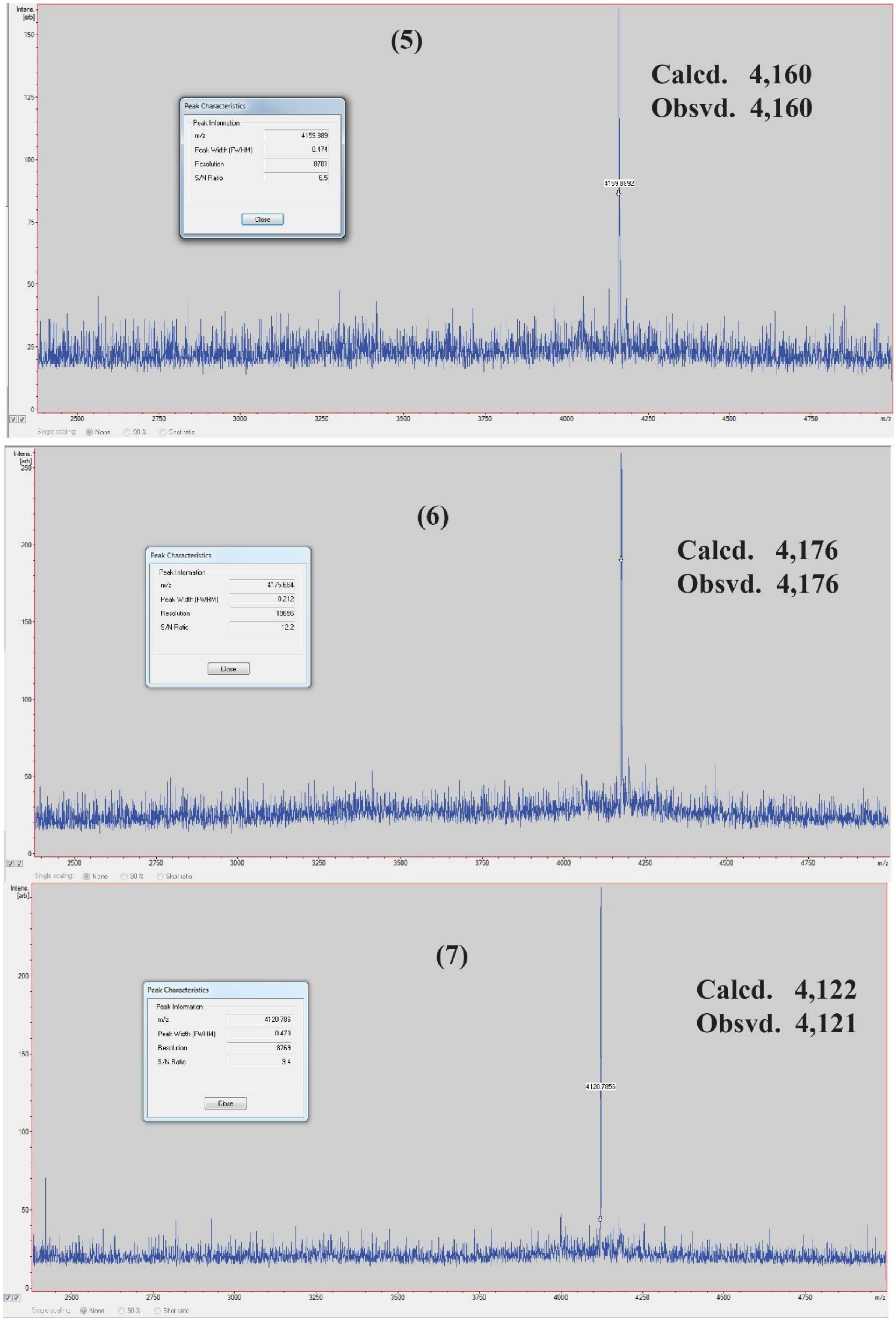

Figure SI 2. MALDI-TOF corresponding to oligonucleotides **5-7**

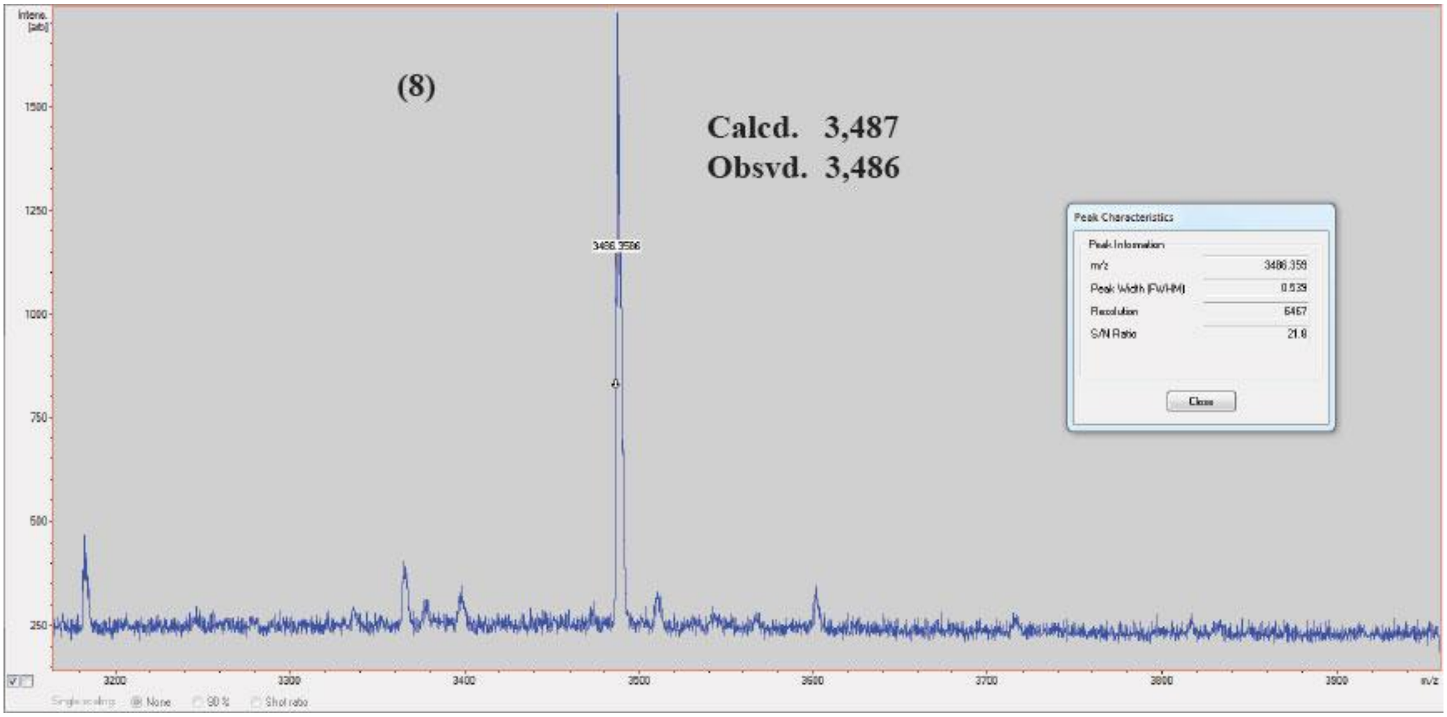

Figure SI 3. MALDI-TOF corresponding to oligonucleotide **8**

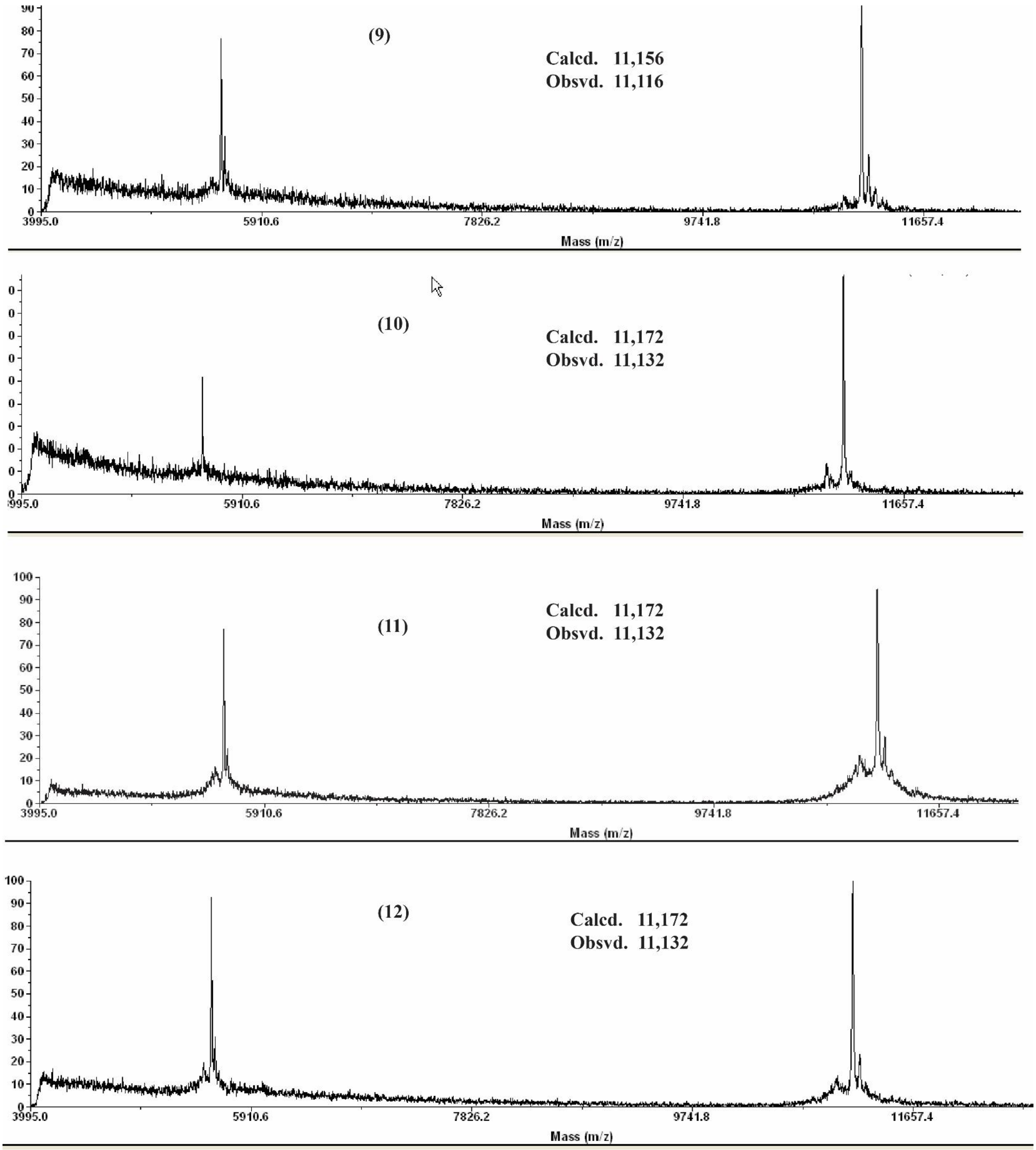

Figure SI 4. MALDI-TOF corresponding to oligonucleotides **9-12**

Job Parameters

Submitted at Mon 16 Sep 2019 01:27:47 PM EDT

RNA at 37°C
$[Na^+] = 0.01$ M, $[Mg^{++}] = 0.005$ M

Computation took 1.0 seconds.

Results

All foldings: ct file, Mac ct, RNAML, PDF

Sequence 1 ΔG = -10.70 ΔH = -98.30 ΔS = -282.44 T_m = 74.9°C Image: PNG PDF Thermodynamic details

Loop Free-Energy Decomposition

Sequence 1, structure 1

ΔG = -10.70 kcal/mol ΔH = -98.30 kcal/mol ΔS = -282.44 e.u. T_m = 74.9°C

**Structural element**	**δδG**	**Information**
External loop	+0.00	0 ss bases & 1 closing helices
Stack	-1.40	External closing pair is G^{1}-C^{33}
Stack	-2.10	External closing pair is G^{2}-U^{32}
Stack	-2.00	External closing pair is C^{3}-G^{31}
**Helix**	-5.50	4 base pairs
Interior loop	+1.50	External closing pair is G^{4}-C^{30}
Stack	-2.90	External closing pair is C^{8}-G^{26}
Stack	-1.80	External closing pair is C^{9}-G^{25}
**Helix**	-4.70	3 base pairs
Bulge loop	+3.50	External closing pair is A^{10}-U^{24}
Stack	-3.40	External closing pair is G^{11}-C^{20}
Stack	-2.90	External closing pair is C^{12}-G^{19}
**Helix**	-6.30	3 base pairs
Hairpin loop	+0.80	Closing pair is C^{13}-G^{18}

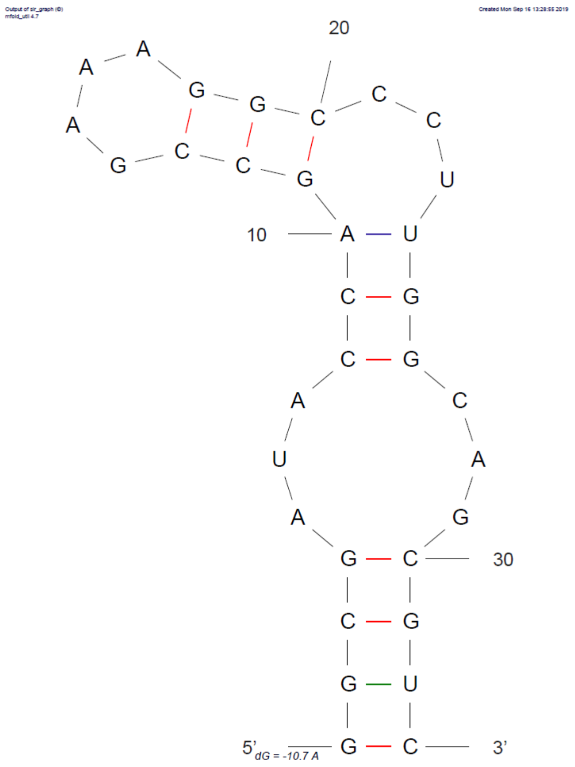

Figure SI 5. UNAFOLD predicted parameters corresponding to canonical aptamer **1**.

Job Parameters

Submitted at Mon 16 Sep 2019 01:32:53 PM EDT

RNA at 37°C
$[Na^{+}] = 0.01$ M, $[Mg^{++}] = 0.005$ M

Computation took 0.0 seconds.

Results

All foldings: ct file, Mac ct, RNAML, PDF

Sequence 1 ΔG = -9.80 ΔH = -76.90 ΔS = -216.35 T_m = 82.3°C Image: PNG PDF Thermodynamic details

Loop Free-Energy Decomposition

Sequence 1, structure 1

ΔG = -9.80 kcal/mol ΔH = -76.90 kcal/mol ΔS = -216.35 e.u. T_m = 82.3°C

Structural element	δδG	Information
External loop	-1.70	7 ss bases & 1 closing helices
Stack	-3.40	External closing pair is G^{2}-C^{27}
Stack	-1.20	External closing pair is C^{3}-G^{26}
Stack	-1.10	External closing pair is G^{4}-U^{25}
**Helix**	-5.70	4 base pairs
Interior loop	+3.10	External closing pair is A^{5}-U^{24}
Stack	-3.40	External closing pair is G^{11}-C^{20}
Stack	-2.90	External closing pair is C^{12}-G^{19}
**Helix**	-6.30	3 base pairs
Hairpin loop	+0.80	Closing pair is C^{13}-G^{18}

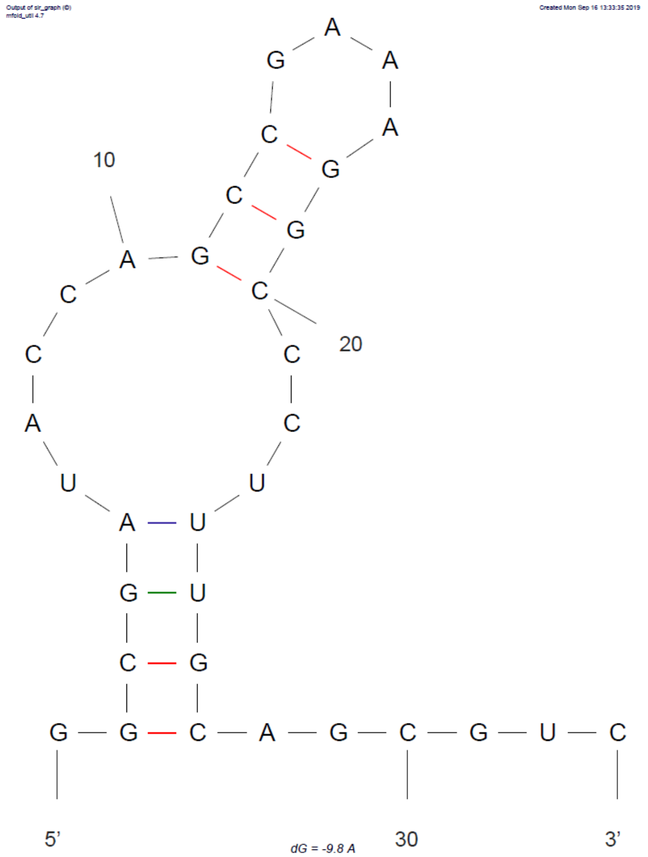

Figure SI 6. UNAFOLD predicted parameters corresponding to modified aptamer **2**, where 8-oxoG at position-25 was substituted with U.

Job Parameters

Submitted at Mon 16 Sep 2019 01:36:09 PM EDT

RNA at 37°C
$[Na^{+}]$ = 0.01 M, $[Mg^{++}]$ = 0.005 M

Computation took 1.0 seconds.

Results

All foldings: ct file, Mac ct, RNAML, PDF

Sequence 1 ΔG = -9.60 ΔH = -99.50 ΔS = -289.86 T_m = 70.1°C Image: PNG PDF Thermodynamic details
Sequence 1 ΔG = -8.70 ΔH = -77.70 ΔS = -222.47 T_m = 76.1°C Image: PNG PDF Thermodynamic details

Loop Free-Energy Decomposition

Sequence 1, structure 1

ΔG = -9.60 kcal/mol ΔH = -99.50 kcal/mol ΔS = -289.86 e.u. T_m = 70.1°C

Structural element	δδG	Information
External loop	-1.70	4 ss bases & 1 closing helices
Stack	-3.40	External closing pair is G^{2}-C^{30}
**Helix**	-3.40	2 base pairs
Bulge loop	+1.90	External closing pair is C^{3}-G^{29}
Stack	-2.30	External closing pair is G^{4}-C^{27}
Stack	-1.00	External closing pair is A^{5}-U^{26}
Stack	-0.80	External closing pair is U^{6}-G^{25}
**Helix**	-4.10	4 base pairs
Interior loop	+3.20	External closing pair is A^{7}-U^{24}
Stack	-3.40	External closing pair is G^{11}-C^{20}
Stack	-2.90	External closing pair is C^{12}-G^{19}
**Helix**	-6.30	3 base pairs
Hairpin loop	+0.80	Closing pair is C^{13}-G^{18}

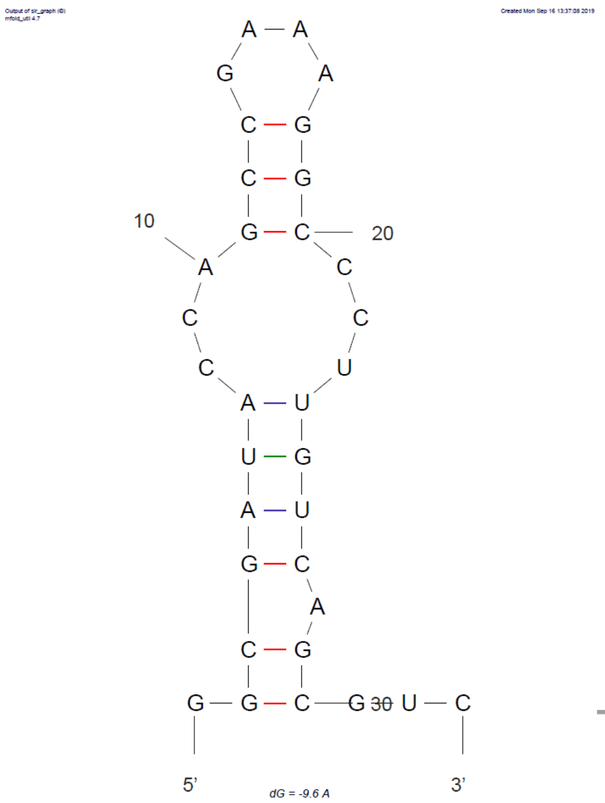

Sequence 1, structure 2

ΔG = -8.70 kcal/mol ΔH = -77.70 kcal/mol ΔS = -222.47 e.u. T_m = 76.1°C

**Structural element**	**δδG**	**Information**
External loop	-1.70	6 ss bases & 1 closing helices
Stack	-1.40	External closing pair is G^1-C^{27}
Stack	-2.10	External closing pair is G^2-U^{26}
Stack	-1.20	External closing pair is C^3-G^{25}
Stack	-1.10	External closing pair is G^4-U^{24}
**Helix**	-5.80	5 base pairs
Interior loop	+4.30	External closing pair is A^5-U^{23}
Stack	-3.40	External closing pair is G^{11}-C^{20}
Stack	-2.90	External closing pair is C^{12}-G^{19}
**Helix**	-6.30	3 base pairs
Hairpin loop	+0.80	Closing pair is C^{13}-G^{18}

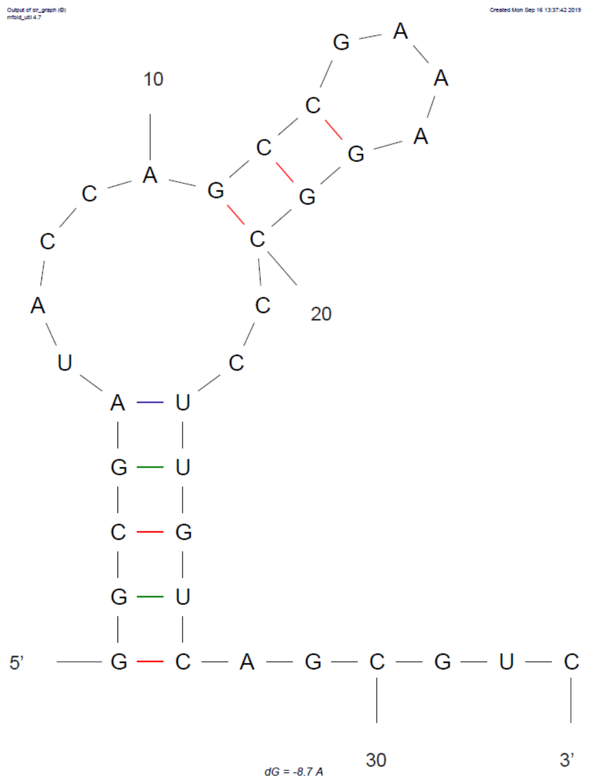

Figure SI 7. UNAFOLD predicted parameters corresponding to modified aptamer **3**, where 8-oxoG at position-26 was substituted with U. Two structures were predicted as plausible candidates.

Job Parameters

Submitted at Mon 16 Sep 2019 01:30:46 PM EDT

RNA at 37°C
$[Na^{+}] = 0.01$ M, $[Mg^{++}] = 0.005$ M

Computation took 0.0 seconds.

Results

All foldings: ct file, Mac ct, RNAML, PDF

Sequence 1 $\Delta G = -8.10$ $\Delta H = -91.30$ $\Delta S = -268.26$ $T_m = 67.2$°C Image: PNG PDF Thermodynamic details

Loop Free-Energy Decomposition

Sequence 1, structure 1

$\Delta G = -8.10$ kcal/mol $\Delta H = -91.30$ kcal/mol $\Delta S = -268.26$ e.u. $T_m = 67.2$°C

**Structural element**	**δδG**	**Information**
External loop	+0.00	0 ss bases & 1 closing helices
Stack	-1.40	External closing pair is G^{1}-C^{33}
Stack	-2.10	External closing pair is G^{2}-U^{32}
Stack	-2.00	External closing pair is C^{3}-G^{31}
**Helix**	-5.50	4 base pairs
Interior loop	+1.50	External closing pair is G^{4}-C^{30}
Stack	-2.90	External closing pair is C^{8}-G^{26}
Stack	-1.80	External closing pair is C^{9}-G^{25}
**Helix**	-4.70	3 base pairs
Interior loop	+2.70	External closing pair is A^{10}-U^{24}
Stack	-2.90	External closing pair is C^{12}-G^{19}
**Helix**	-2.90	2 base pairs
Hairpin loop	+0.80	Closing pair is C^{13}-G^{18}

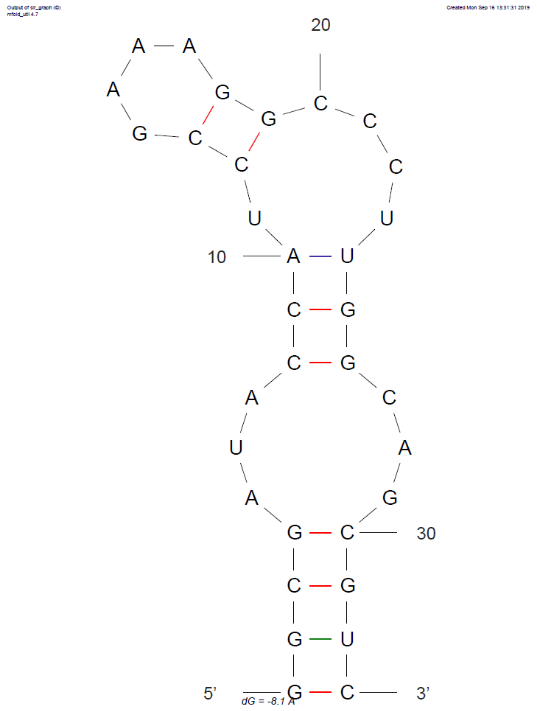

Figure SI 8. UNAFOLD predicted parameters corresponding to modified aptamer **3**, where 8-oxoG at position-11 was substituted with U.

Loop Free-Energy Decomposition

Sequence 1, structure 1

ΔG = -6.50 kcal/mol ΔH = -45.50 kcal/mol ΔS = -125.75 e.u. T_m = 88.7°C

Structural element	δδG	Information
External loop	-1.00	3 ss bases & 1 closing helices
Stack	-3.40	External closing pair is G^{2}-C^{11}
Stack	-2.90	External closing pair is C^{3}-G^{10}
**Helix**	-6.30	3 base pairs
Hairpin loop	+0.80	Closing pair is C^{4}-G^{9}

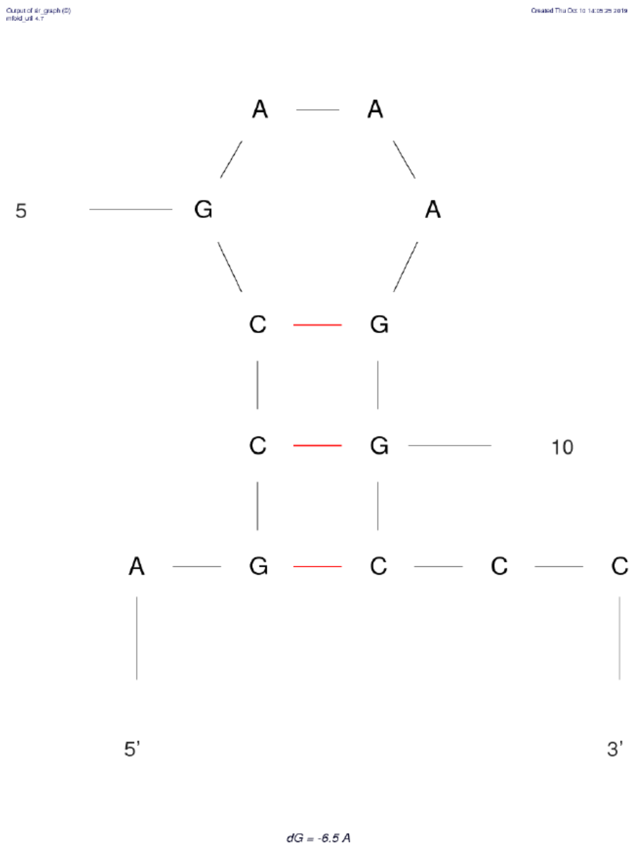

Figure SI 9. UNAFOLD predicted parameters corresponding to hairpin **5.**

Loop Free-Energy Decomposition

Sequence 1, structure 1

ΔG = -2.60 kcal/mol ΔH = -29.80 kcal/mol ΔS = -87.70 e.u. T_m = 66.6°C

**Structural element**	**δδG**	**Information**
External loop	-0.50	5 ss bases & 1 closing helices
Stack	-2.90	External closing pair is C^3-G^{10}
**Helix**	-2.90	2 base pairs
Hairpin loop	+0.80	Closing pair is C^4-G^9

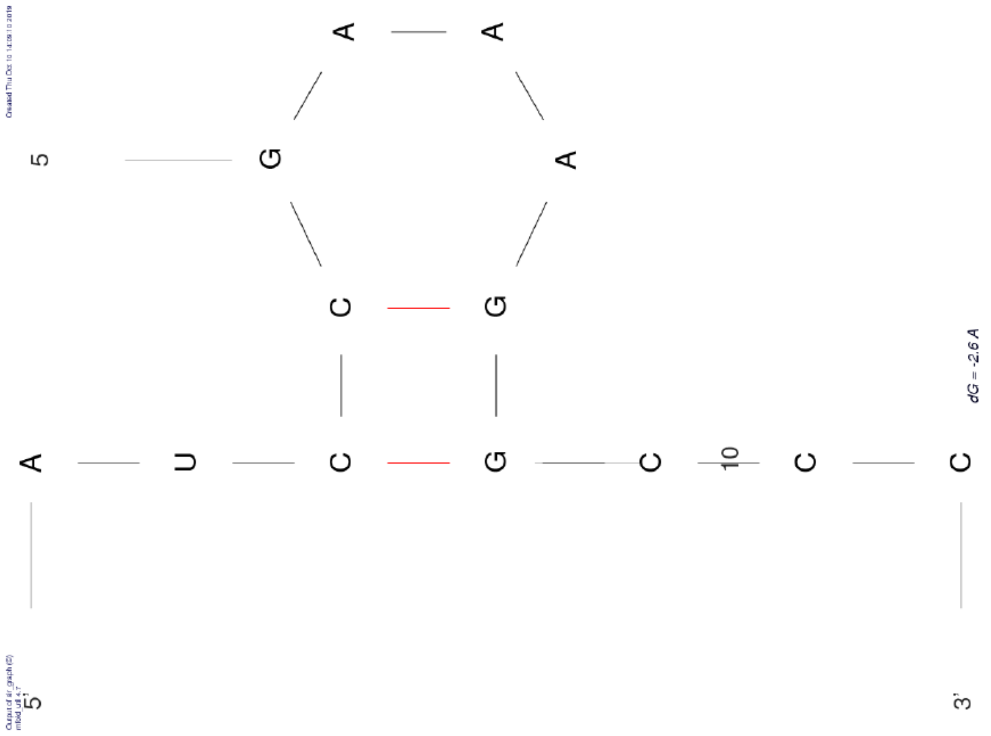

Figure SI 10. UNAFOLD predicted parameters corresponding to hairpin **6,** where 8-oxoG within the stem was substituted with U.

Loop Free-Energy Decomposition

Sequence 1, structure 1

ΔG = -2.50 kcal/mol ΔH = -28.40 kcal/mol ΔS = -83.51 e.u. T_m = 66.9°C

**Structural element**	**δδG**	**Information**
External loop	-0.40	3 ss bases & 1 closing helices
Stack	-2.90	External closing pair is C^1-G^8
**Helix**	-2.90	2 base pairs
Hairpin loop	+0.80	Closing pair is C^2-G^7

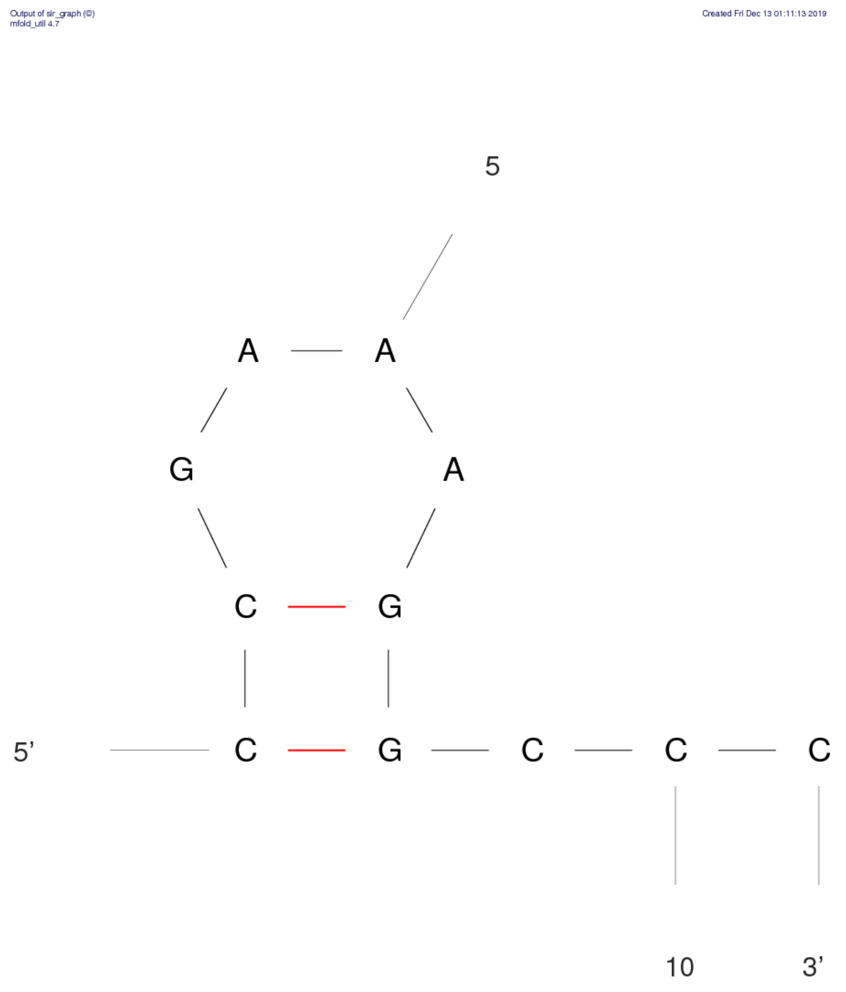

Figure SI 11. UNAFOLD predicted parameters corresponding to hairpin **8,** where two nucleotides from the 5’-end were omitted.

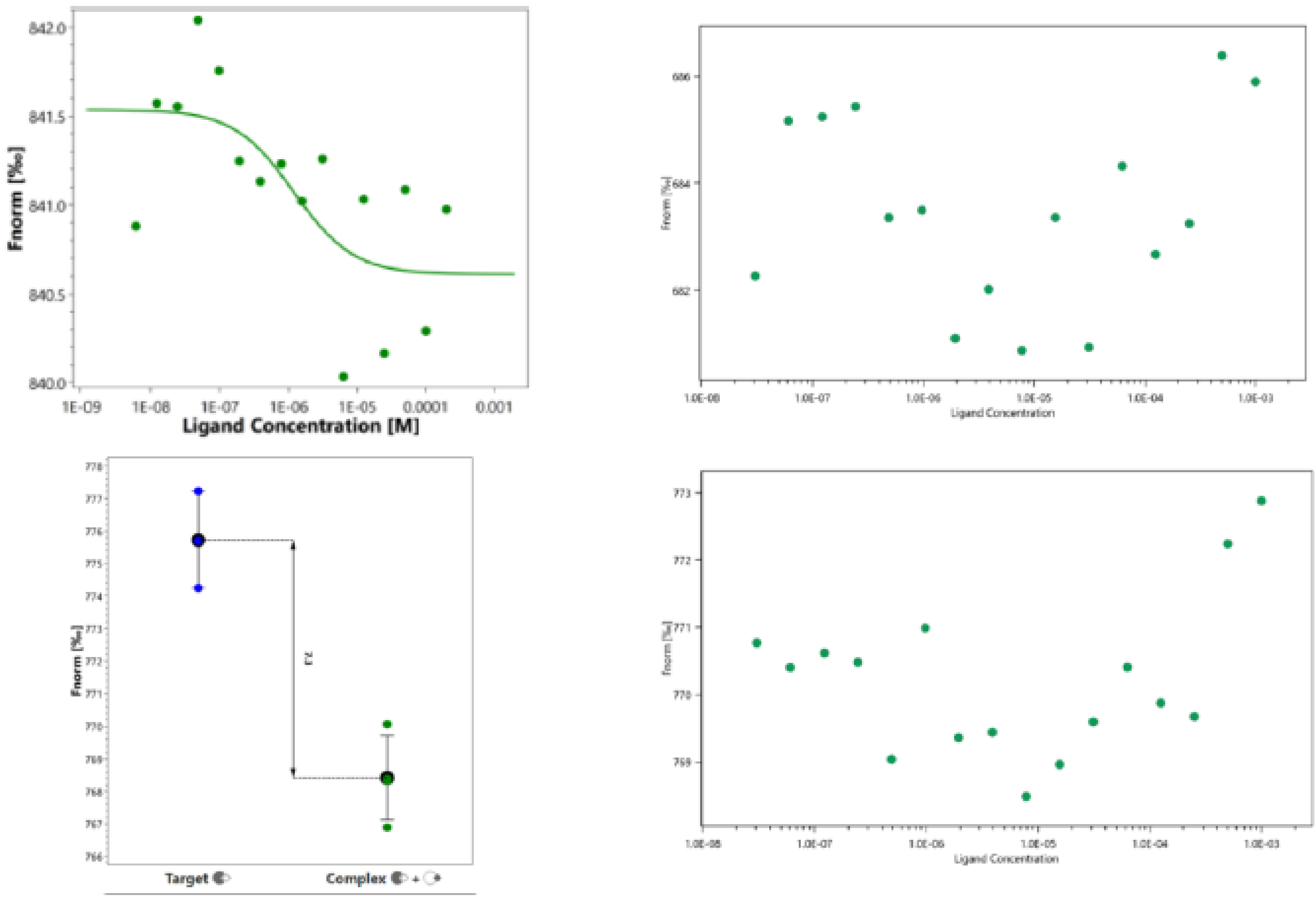

Figure SI 12. MST of RNA **9** with theobromine (not binding, top left); binding of **10** with theobromine (bottom left); and RNAs **11** (top right) and **12** (bottom right) not binding to theobromine.

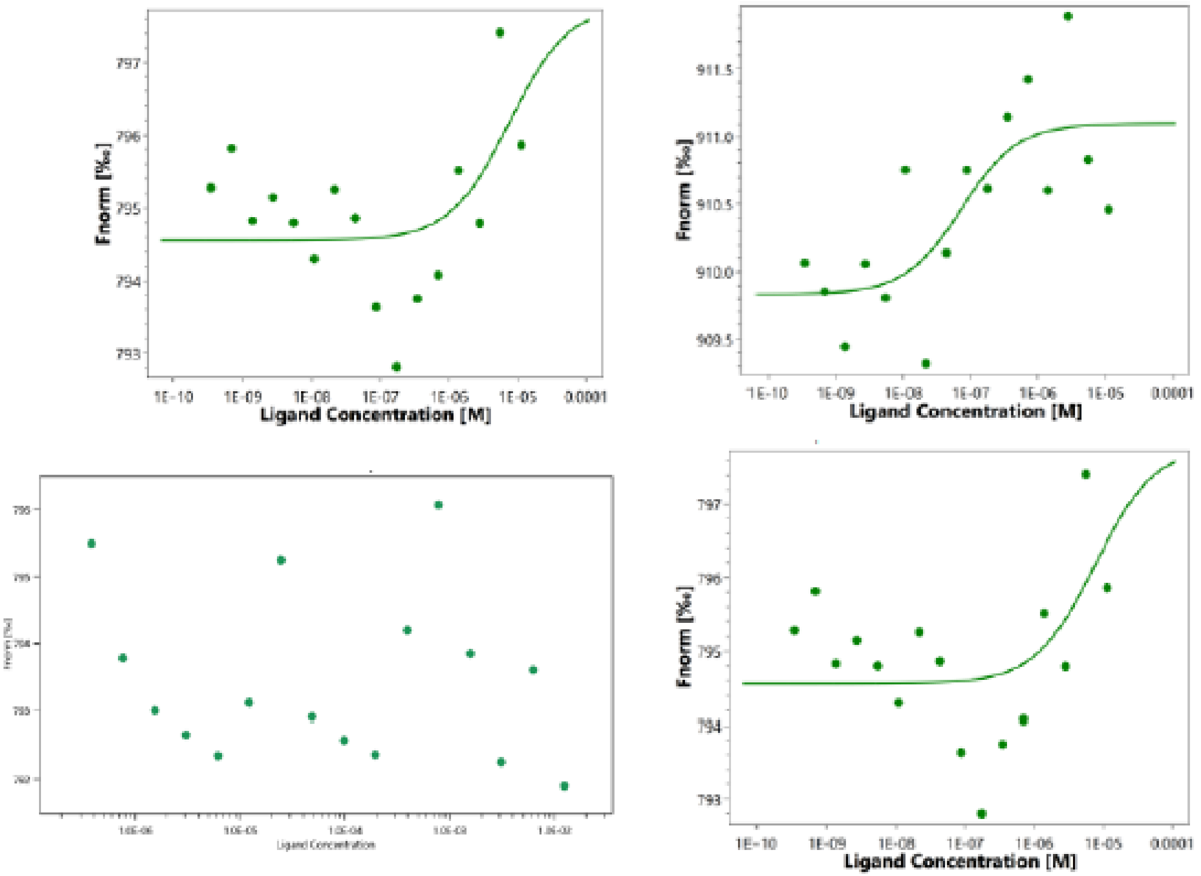

Figure SI 13. MST of RNA **9** (top left), **10** (top right), **11** (bottom left) and **12** (bottom right) not binding to caffeine.

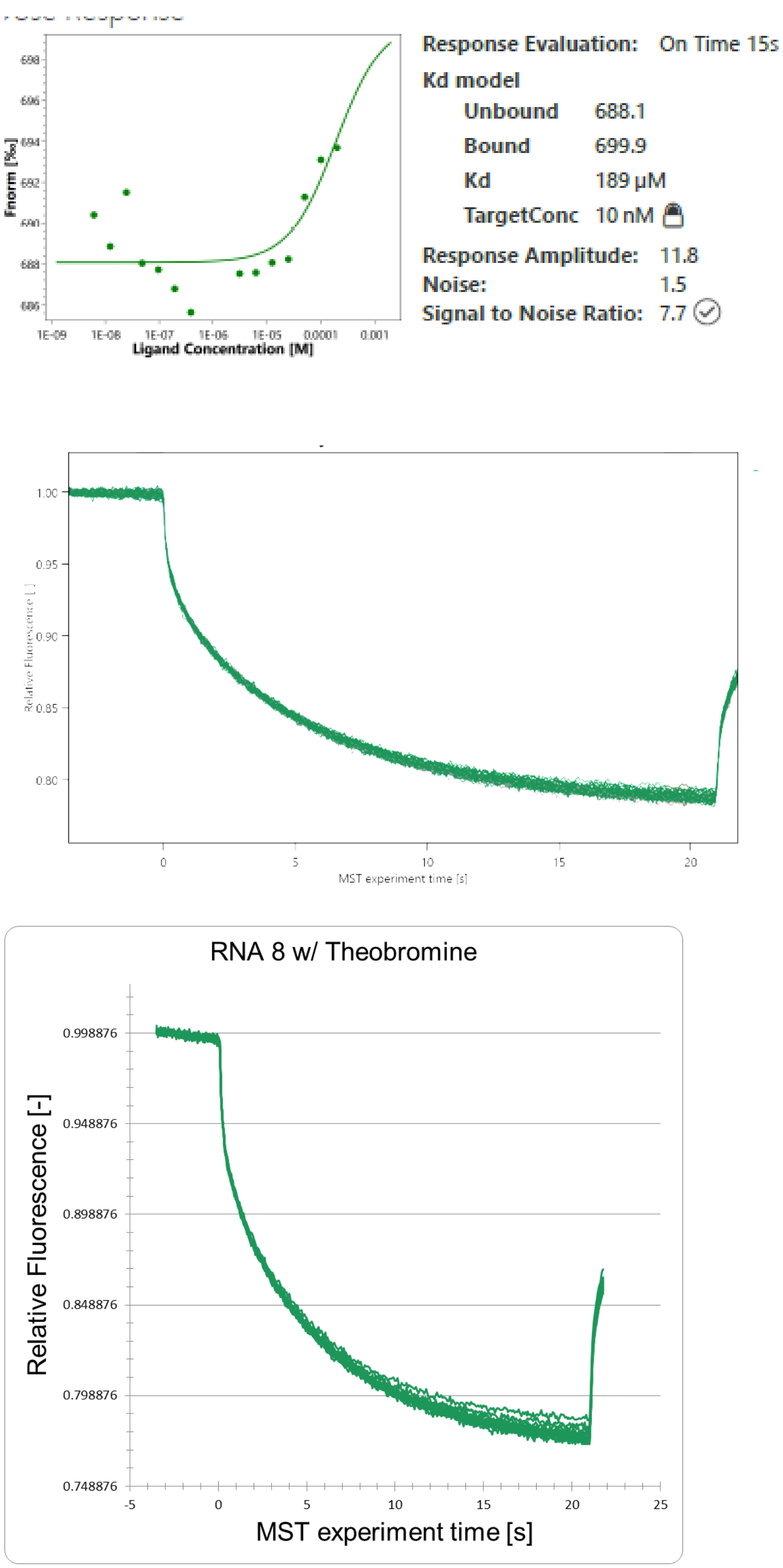

Figure SI 14. MST of RNA **10** binding with theobromine in a PBS buffer system (top); and binding checks of the same RNA-xanthine pair.

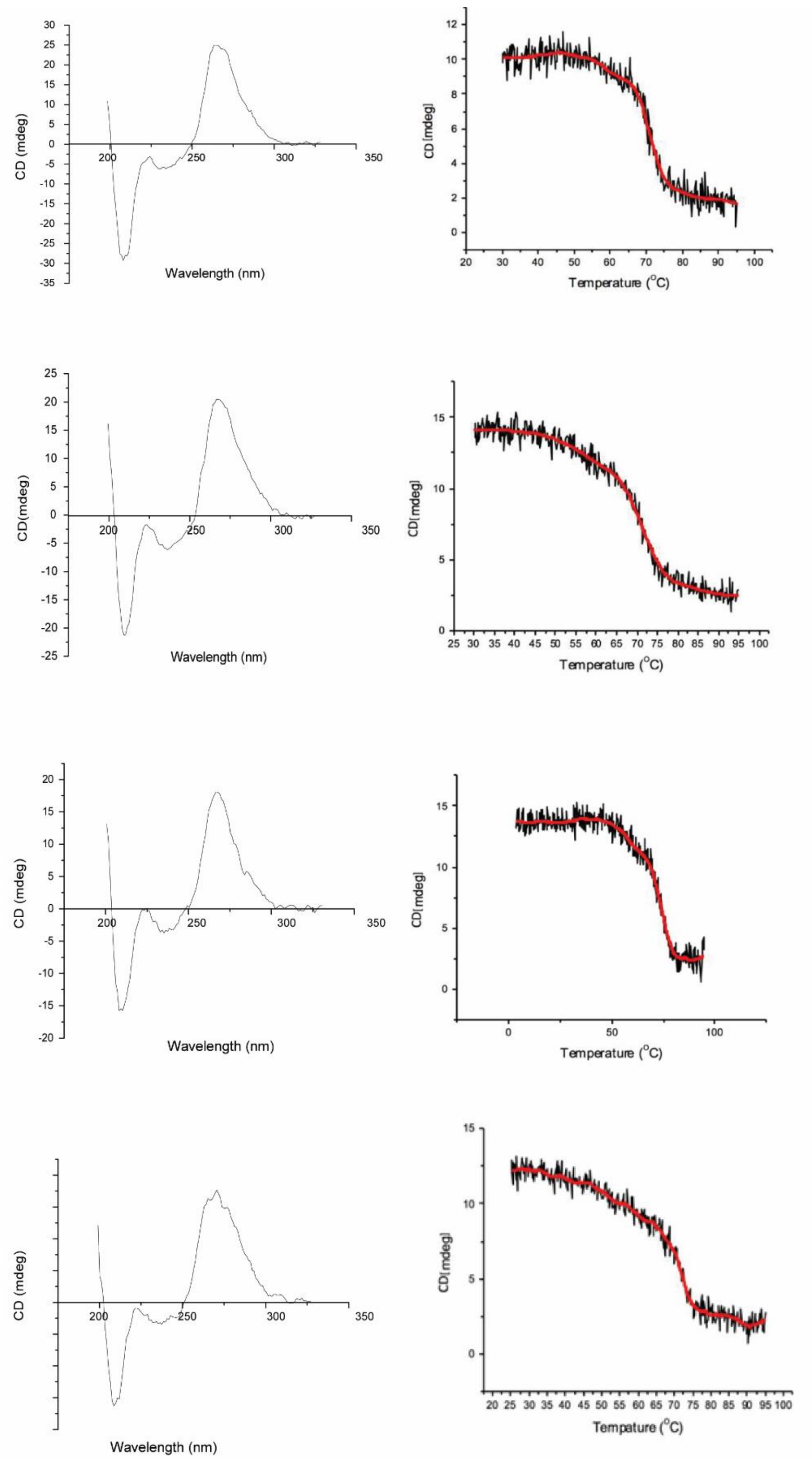

Figure SI 15. CD spectra and T_m curves corresponding to RNAs **7-10** (top to bottom).

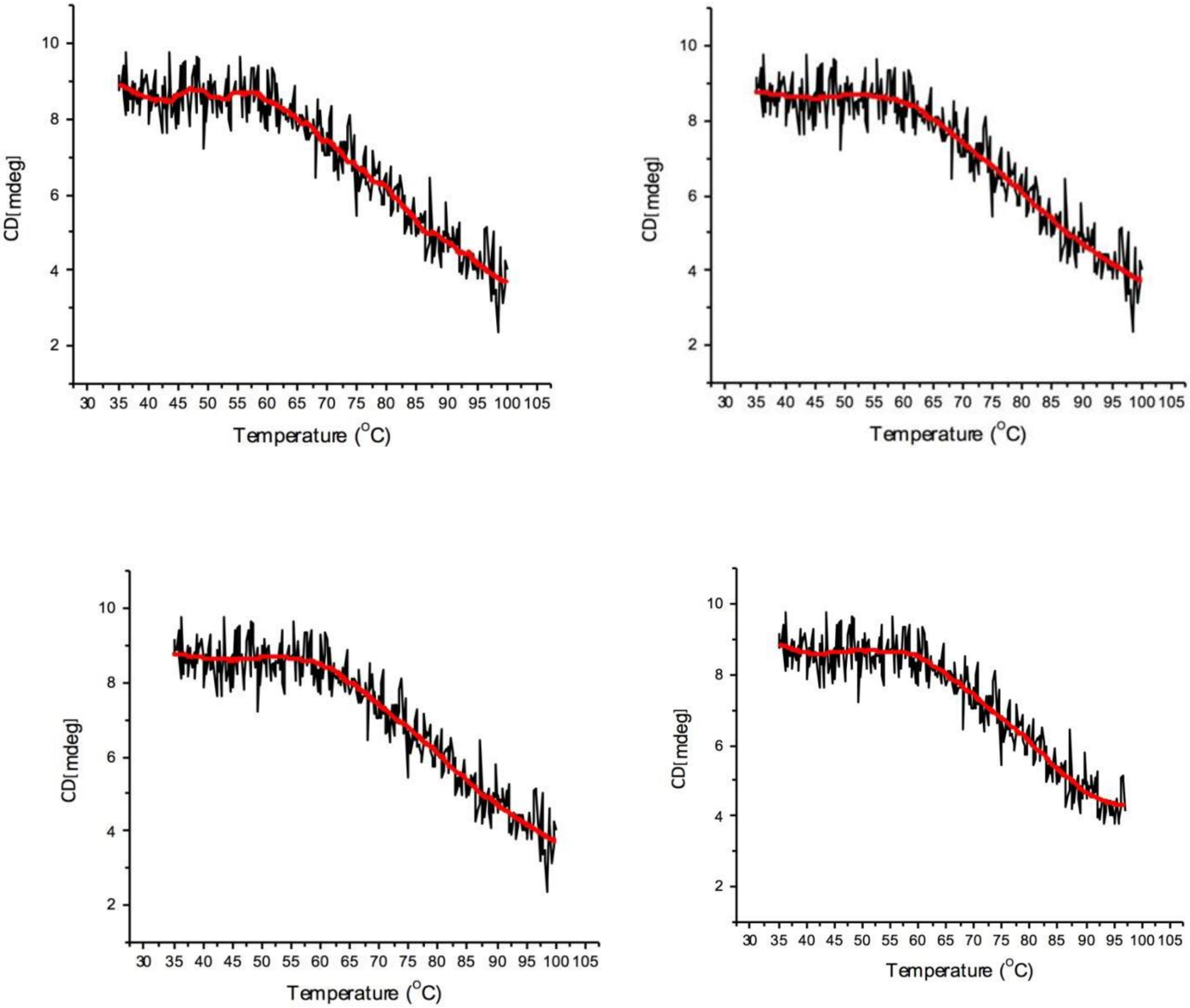

Figure SI 16. T_m curves corresponding to RNAs **9-10** (top) and **11-12** (top) in TBS buffer, pH 7.6.

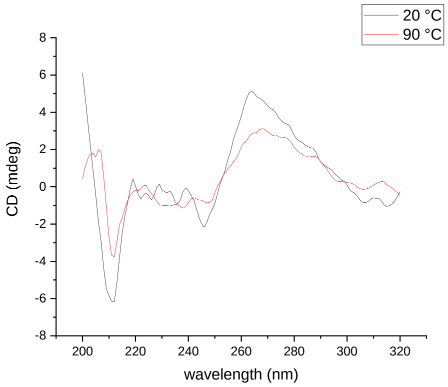

Figure 17. Thermal transition of RNA **5** as measured by circular dichroism

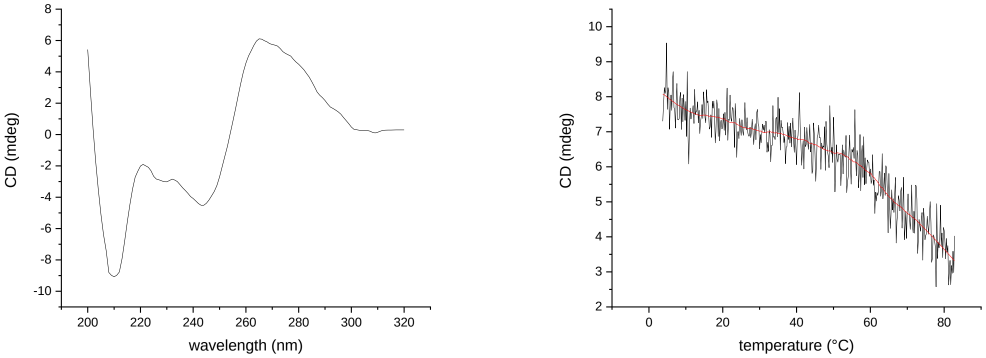

Figure 18. Typical CD spectrum (left) and thermal denaturation curve (right) of RNA **5**

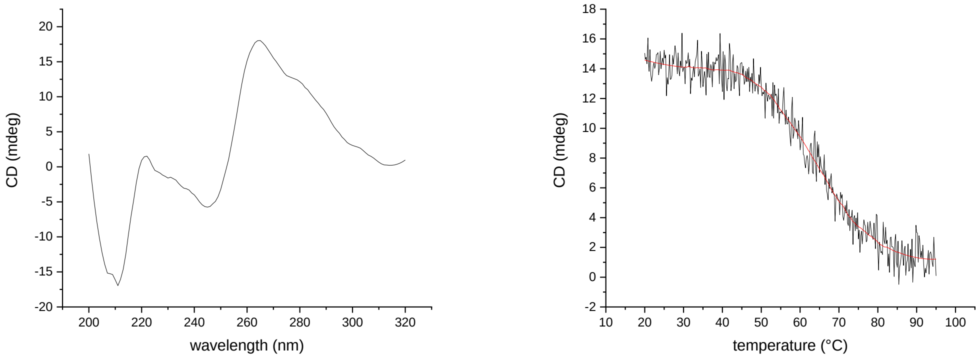

Figure 19. Typical CD spectrum (left) and thermal denaturation curve (right) of RNA **6**

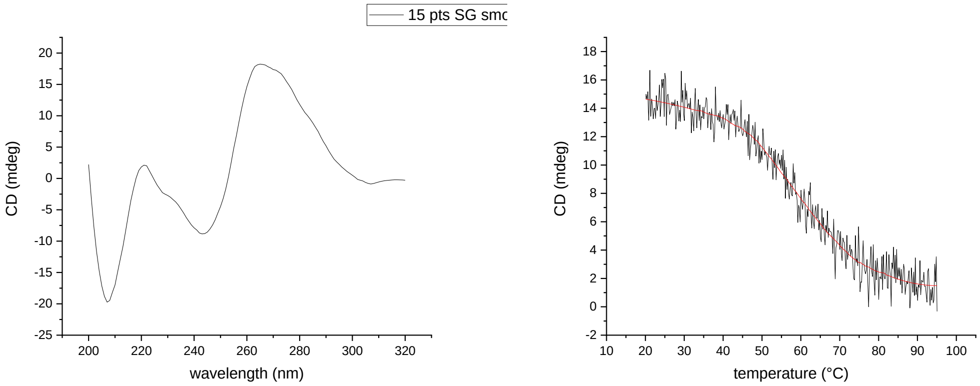

Figure 20. Typical CD spectrum (left) and thermal denaturation curve (right) of RNA **7**

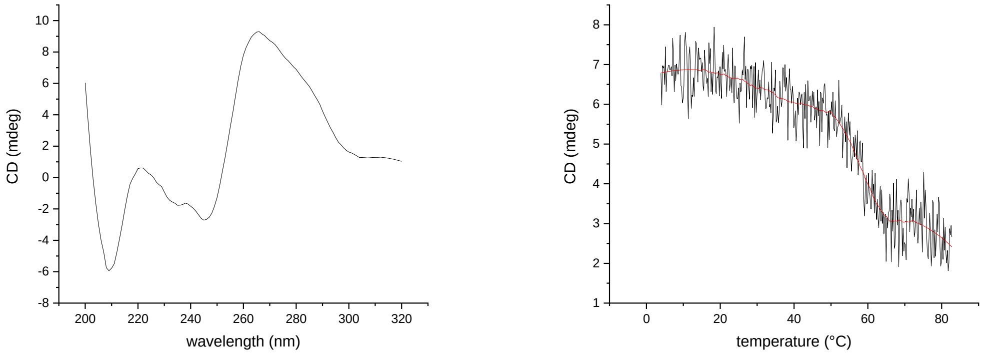

Figure 21. Typical CD spectrum (left) and thermal denaturation curve (right) of RNA **8**

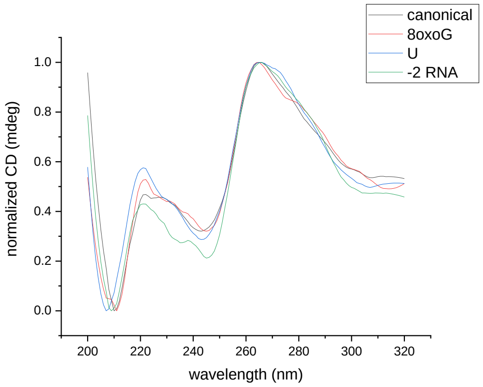

Figure 22. Normalized CD overlay between all 4 hairpin models **5-8**.

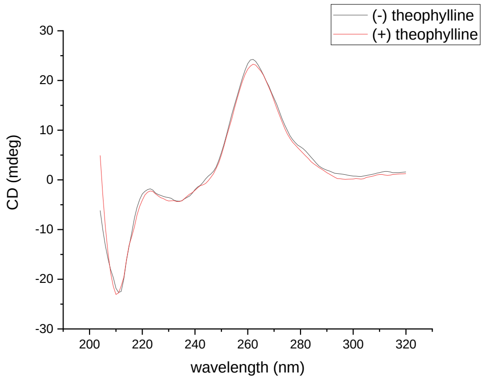

Figure 23. CD binding study of RNA **9** in presence and absence of 1 mM theophylline in 1x TBS

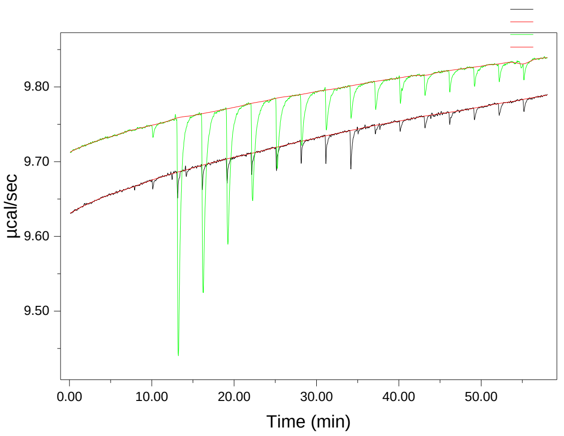

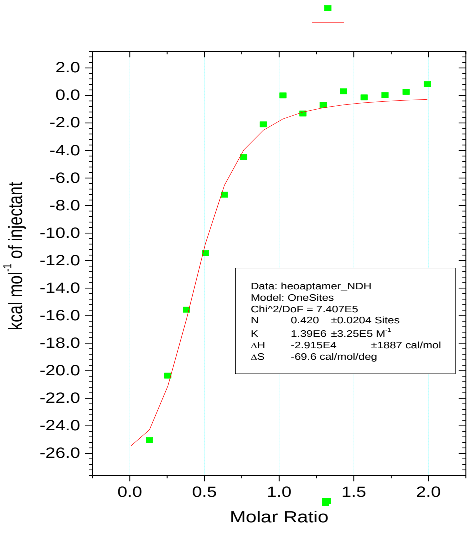

K$_d$ = 0.72 µM

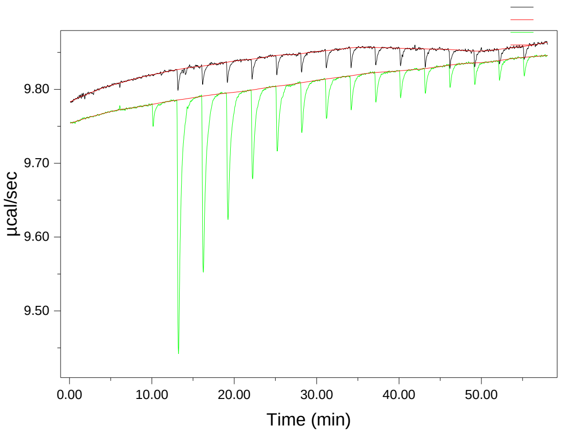

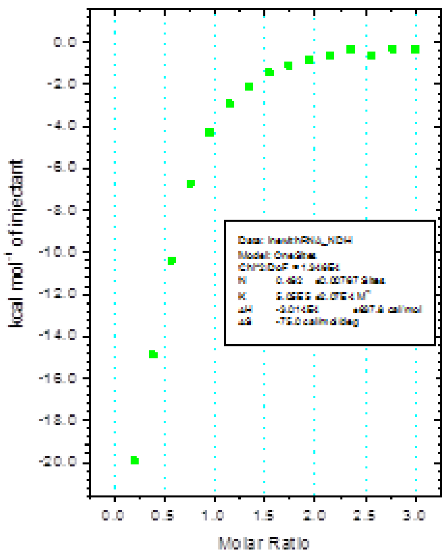

K$_d$ = 1.98 µM

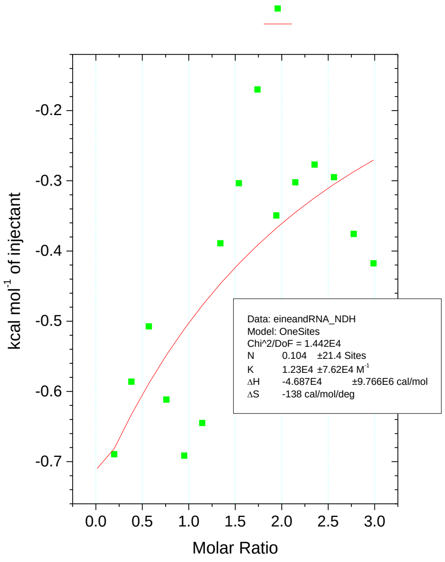

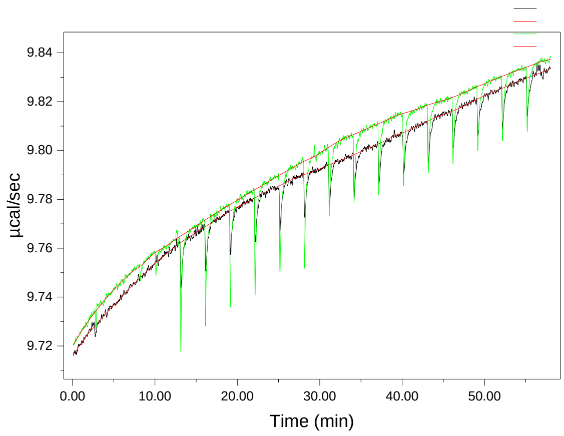

K$_d$ = NA

Figure 24. ITC of RNA **1** with theophylline (top and middle) and with caffeine (bottom). Conditions were carried out as previously reported.[(1)]

References:

(1) Lee, S. W., Zhao, L., Pardi, A., Xia, T. *Biochemistry*, **2010**, *49*, 2943-2951.
